# Cryo-EM structure of the SARS coronavirus spike glycoprotein in complex with its host cell receptor ACE2

**DOI:** 10.1371/journal.ppat.1007236

**Published:** 2018-08-13

**Authors:** Wenfei Song, Miao Gui, Xinquan Wang, Ye Xiang

**Affiliations:** 1 The Ministry of Education Key Laboratory of Protein Science, Beijing Advanced Innovation Center for Structural Biology, Collaborative Innovation Center for Biotherapy, School of Life Sciences, Tsinghua University, Beijing, China; 2 Center for Infectious Disease Research, Collaborative Innovation Center for Diagnosis and Treatment of Infectious Diseases, Beijing Advanced Innovation Center for Structural Biology, Department of Basic Medical Sciences, School of Medicine, Tsinghua University, Beijing, China; 3 Collaborative Innovation Center for Biotherapy, State Key Laboratory of Biotherapy and Cancer Center, West China Hospital, West China Medical School, Sichuan University, Chengdu, Sichuan, China; University of North Carolina at Chapel Hill, UNITED STATES

## Abstract

The trimeric SARS coronavirus (SARS-CoV) surface spike (S) glycoprotein consisting of three S1-S2 heterodimers binds the cellular receptor angiotensin-converting enzyme 2 (ACE2) and mediates fusion of the viral and cellular membranes through a pre- to postfusion conformation transition. Here, we report the structure of the SARS-CoV S glycoprotein in complex with its host cell receptor ACE2 revealed by cryo-electron microscopy (cryo-EM). The complex structure shows that only one receptor-binding domain of the trimeric S glycoprotein binds ACE2 and adopts a protruding “up” conformation. In addition, we studied the structures of the SARS-CoV S glycoprotein and its complexes with ACE2 in different *in vitro* conditions, which may mimic different conformational states of the S glycoprotein during virus entry. Disassociation of the S1-ACE2 complex from some of the prefusion spikes was observed and characterized. We also characterized the rosette-like structures of the clustered SARS-CoV S2 trimers in the postfusion state observed on electron micrographs. Structural comparisons suggested that the SARS-CoV S glycoprotein retains a prefusion architecture after trypsin cleavage into the S1 and S2 subunits and acidic pH treatment. However, binding to the receptor opens up the receptor-binding domain of S1, which could promote the release of the S1-ACE2 complex and S1 monomers from the prefusion spike and trigger the pre- to postfusion conformational transition.

## Author summary

The global outbreak of SARS in 2002–2003 was caused by infection by a human coronavirus, SARS-CoV. Although the virus has been extensively studied with regard to epidemiology, virology, clinical features and other aspects, there are still no approved antiviral drugs and vaccines to treat and prevent infections of SARS-CoV. The spike (S) glycoprotein of the coronavirus, responsible for host cell attachment and mediating host cell membrane and viral membrane fusion during infection, is key to the viral life cycle and a major target for antiviral drugs and vaccines. In this study, we report the structures of different conformational states of the SARS-CoV S glycoprotein during virus entry. Specifically, we found that the S glycoprotein retains the prefusion trimer structure after trypsin cleavage and low-pH treatment. Additionally, binding with host cell receptor ACE2 promotes the release of S1 subunits from the S trimer and triggers the pre- to postfusion conformational transition. Our results provide new insights for understanding the mechanisms involved in coronavirus S glycoprotein-mediated virus entry.

## Introduction

Coronaviruses are a family of large, enveloped, positive-stranded RNA viruses that cause upper respiratory, gastrointestinal and central nervous system diseases in humans and other animals [1]. Human coronaviruses HCoV-OC43, HCoV-229E, HCoV-NL63 and HCoV-HKU1 circulate in humans and cause mild respiratory diseases [2]. However, the outbreak of SARS-CoV in 2002 and MERS-CoV in 2012 showed that coronaviruses can cross the species barrier and emerge as highly pathogenic viruses [3]. The high fatality rate and wide spread of these new emerging coronaviruses indicate that they are a severe threat to global health.

The spike (S) glycoprotein of the coronavirus is a class I viral fusion protein located on the outer envelope of the virion that plays a critical role in viral infection by recognizing host cell receptors and mediating fusion of the viral and cellular membranes [4]. The coronavirus S glycoprotein is synthesized as a precursor protein consisting of ~1,300 amino acids that is then cleaved into an amino (N)-terminal S1 subunit (~700 amino acids) and a carboxyl (C)-terminal S2 subunit (~600 amino acids). Three S1/S2 heterodimers assemble to form a trimer spike protruding from the viral envelope. The S1 subunit contains a receptor-binding domain (RBD), while the S2 subunit contains a hydrophobic fusion peptide and two heptad repeat regions. Triggered by receptor binding, proteolytic processing and/or acidic pH in the cellular compartments, the class I viral fusion protein undergoes a transition from a metastable prefusion state to a stable postfusion state during infection, in which the receptor-binding subunit is cleaved, and the fusion subunit undergoes large-scale conformational rearrangements to expose the hydrophobic fusion peptide, induce the formation of a six-helix bundle, and bring the viral and cellular membranes close for fusion [5]. Coronavirus S glycoprotein features two distinct protease cleavage sites. The S1/S2 cleavage site of the SARS-CoV S glycoprotein is located after residue 667 of the precursor protein, whereas the S2’ cleavage site of the SARS-CoV S glycoprotein is on the S2 subunit and is 130 amino acids from the N terminus of the S2 subunit [6–8]. The S1/S2 cleavage site is located in a flexible loop of residues 660–675 that is completely exposed in the prefusion S1-S2 trimer spike. The S2’ cleavage site of the SARS-CoV S glycoprotein is highly conserved among coronaviruses and is completely buried in the prefusion SARS-CoV S glycoprotein [6–8]. Cleavage of the S2’ site by host cell proteases is required for successful infection by SARS-CoV [8, 9]. However, the mechanisms involved in exposure and cleavage of the S2’ cleavage site are not well understood. Structural biology studies, especially recent cryo-electron microscopy (cryo-EM) studies, have advanced our understanding of the role of the coronavirus S glycoprotein in virus entry. S glycoprotein structures in the prefusion state have been reported for members from the Alphacoronavirus genus (HCoV-NL63), Betacoronavirus genus (mouse hepatitis virus (MHV), HKU1, SARS-CoV and MERS-CoV), Gammacoronavirus genus (IBV), and Deltacoronavirus genus (PdCoV) [7, 10–17]. Prefusion S glycoproteins adopt a similar mushroom-like homo-trimer architecture, of which the stem is mainly composed of three S2 subunits, and the top cap consists of three interwoven S1 subunits. A recently reported cryo-EM structure of the MHV S glycoprotein in its postfusion state shows an elongated cone-shaped structure that is significantly different from the prefusion structure and suggests that dramatic conformational changes occur during the prefusion to postfusion transition [18].

The prefusion SARS-CoV S1 subunit is structurally organized into four distinct domains: NTD, CTD1, CTD2 and CTD3 [13]. Among these, CTD1 is the receptor-binding domain, and one CTD1 in the trimer adopts an “up” conformation as a prerequisite for the binding of SARS-CoV to the cellular receptor angiotensin-converting enzyme 2 (ACE2) [13]. Similar observations of a protruding “up” CTD1 have also been reported for MERS-CoV S glycoproteins [7, 16]. Although crystal structures of CTD1 in complex with ACE2 have been reported [19, 20], the structure of the trimeric coronavirus S glycoprotein in complex with the cellular receptor has not been reported. The mechanisms involved in the conformational changes of the S glycoprotein during coronavirus infection, especially for the highly pathogenic SARS-CoV and MERS-CoV, are not completely understood.

Here, we report the SARS-CoV S glycoprotein structures observed by cryo-EM in different stages, including the SARS-CoV S glycoprotein structures in ACE2-free and ACE2-bound states after trypsin cleavage of the S1/S2 site and acidic pH treatment. We also observed and characterized the disassociated S1-ACE2 complex and the postfusion S2 trimeric core. These results collectively enrich our understanding of the SARS-CoV S glycoprotein and its conformational rearrangements during virus entry.

## Results

### Cryo-EM structure determination

By following a similar procedure to those described in previous studies [13], we prepared a SARS-CoV S glycoprotein mutant, of which the S1/S2 cleavage site was impaired by mutating Arg667 to Ala (S1A Fig). The mutated S glycoprotein was mixed with ACE2 at a molar ratio of approximately 1:4, and the mixture was further purified by gel-filtration chromatography to isolate the complex. Fractions of the two elution peaks were collected by gel-filtration chromatography, and the fractions containing the complex were subjected to EM analysis. Cryo-EM analysis of the complex sample showed two major types of particles: the S glycoprotein alone and S glycoprotein bound to ACE2 (S1B Fig). However, the percentage of the S-ACE2 complex particle was less than 7%.

We then prepared wild-type S glycoprotein in insect cells. SDS-page gel analysis of the purified sample showed that only a trace of the S glycoprotein had been proteolytically processed by host proteases. By following a published protocol [21], the sample was further treated with trypsin that completely cleaved the intact S glycoprotein into the S1 and S2 subunits (S2A Fig). Native page gel analysis of the cleaved S glycoprotein sample showed that the S1 and S2 subunits remained associated after cleavage (S2B Fig). Cryo-EM analysis of the cleaved S glycoprotein showed an intact trimer in both the neutral-pH (pH 7.2) and the low-pH (pH 5.6) buffer. The maps are consistent with the prefusion S trimer structure previously determined using the Arg667Ala mutant (S2C–S2F Fig) [13]. These results indicate that cleavage of the S1/S2 site does not significantly change the structure, which is consistent with the conclusions of previous biochemical and structural studies [21].

The S-ACE2 complex was then prepared using trypsin-cleaved and low-pH-treated S glycoprotein and ACE2. Cryo-EM analysis of the complex showed a significant increase in complex particles (49% of the total particles) (S3 Fig, S1 Table). After 3D classification and refinement, three conformational states of the S-ACE2 complex were captured and determined at resolutions of 5.4 Å, 4.2 Å and 4.5 Å. Two conformational states of the ACE2-free S glycoprotein were also determined at resolutions of 3.6 Å and 3.9 Å (S3 and S4 Figs, S2 Table).

### Overall structure of SARS-CoV S trimer bound with one ACE2 receptor

**Fig 1:**
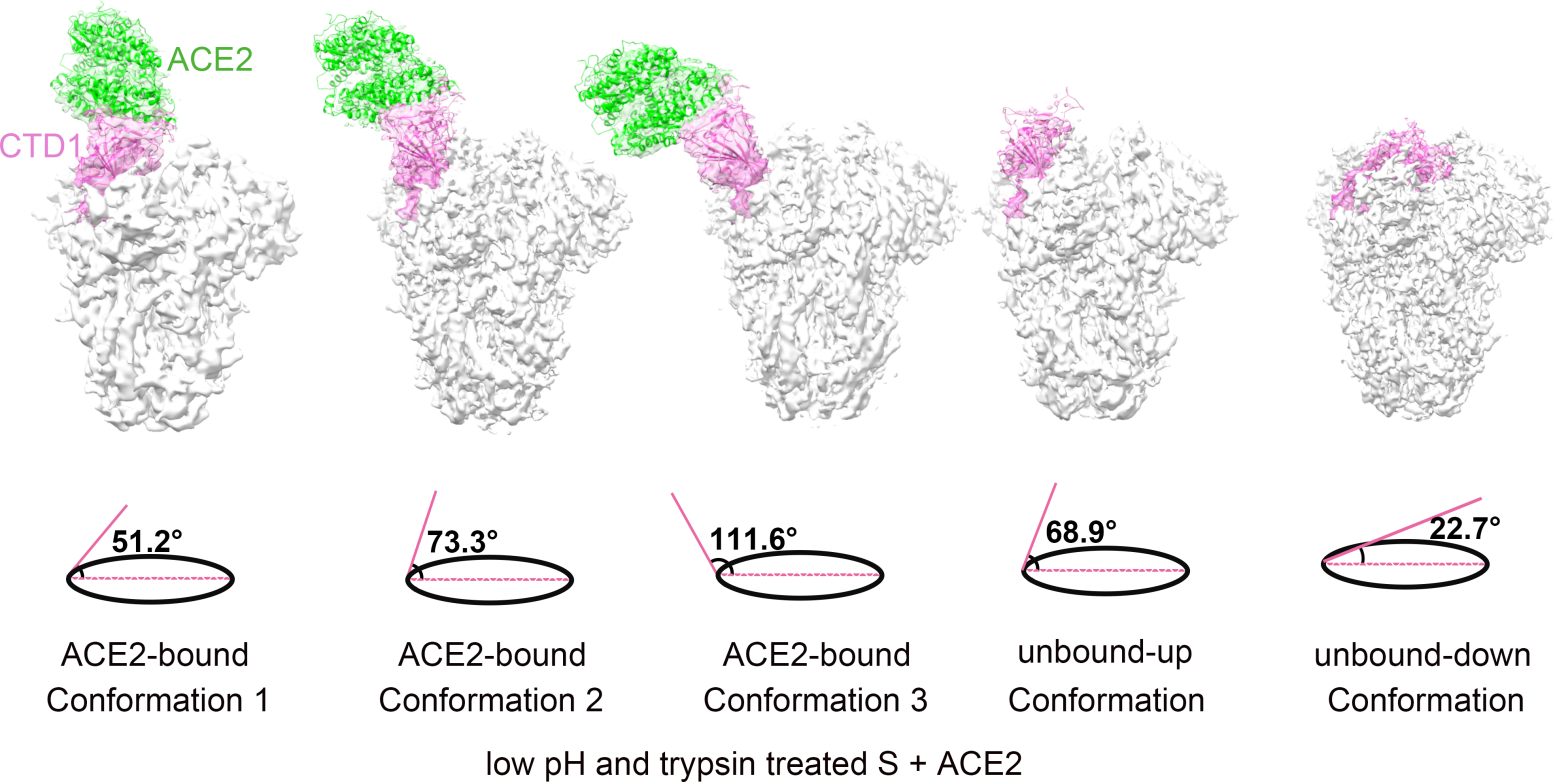
Density maps of the ACE2-bound and ACE2-free SARS-CoV spikes. Surface shadowed diagrams of the ACE2-bound conformation 1, the ACE2-bound conformation 2, the ACE2-bound conformation 3, unbound-up and unbound-down conformations of the SARS-CoV S glycoprotein after trypsin cleavage and low pH treatment. One CTD1 is colored pink and the bound ACE2 is colored green. The angle between the long axes of the CTD1 and the horizontal plane is shown at the bottom of each conformation.

Our cryo-EM analysis of the SARS-CoV S glycoprotein and ACE2 complex sample captured three ACE2-bound and two ACE2-free conformational states of the trypsin-cleaved and low-pH-treated SARS-CoV S glycoprotein. The ACE2-bound states showed that the SARS-CoV S glycoprotein binds one ACE2 receptor utilizing only the “up” CTD1 (Fig 1, S5 and S6 Figs). The “up” CTD1 and the bound ACE2 are flexible, as shown by a 3D classification analysis that yielded three major ACE2-bound conformational states, in which the “up” CTD1s had different “up” angles (the angle between the long axes of the “up” CTD1 and the horizontal plane) of 51.2°, 73.3° and 111.6° (Fig 1A–1C). The structure of one of the two ACE2-free states had one CTD1 in the “up” position and was determined at a resolution of 3.9 Å (Fig 1D). This conformation was designated as the unbound-up conformation, which is ready for receptor binding and represents a receptor-binding active state. The structure of the other ACE2-free state was determined at a resolution of 3.6 Å with C3 symmetry imposed (Fig 1E). This ACE2-free state had all three CTD1s in the “down” position and was designated as the unbound-down conformation that is not accessible for receptor binding. We also prepared a complex consisting of ACE2 and the trypsin-cleaved SARS-CoV S glycoprotein without low-pH treatment. Cryo-EM analysis showed similar ACE2-bound, unbound-up and unbound-down conformations (S7 Fig). Collectively, these results showed different conformational states of the SARS-CoV S glycoprotein and confirmed that the “up” conformation of CTD1 is required for ACE2 binding. Decreasing the pH does not induce significant conformational changes of the S glycoprotein, even after complete cleavage of the precursor S glycoprotein. Interestingly, only one of the three CTD1s in the S glycoprotein was observed in the “up” position in all analyzed structures.

### Structural comparisons of the ACE2-bound and ACE2-free spikes

Simultaneous observation of the ACE2-bound, unbound-up and unbound-down conformations of the S glycoprotein in the trypsin- and low-pH-treated sample provided an opportunity to investigate possible conformational changes induced by receptor binding. Structural comparisons of the CTD1s from different ACE2-free conformational states showed that the “up” angles of the CTD1s were between 50° and 70°, while the angle for the CTD1s in the “down” position was approximately 22.7° (Fig 1D and 1E) [13]. However, the “up” angles of the CTD1s from the ACE2-bound conformations were in the range of 50° to 111.6°. Approximately 19% of the ACE2-bound particles had their CTD1s open to 111.6° (Fig 1C), which was not observed in any of the ACE2-free conformational states. This result indicates that receptor binding can open up CTD1.

**Fig 2:**
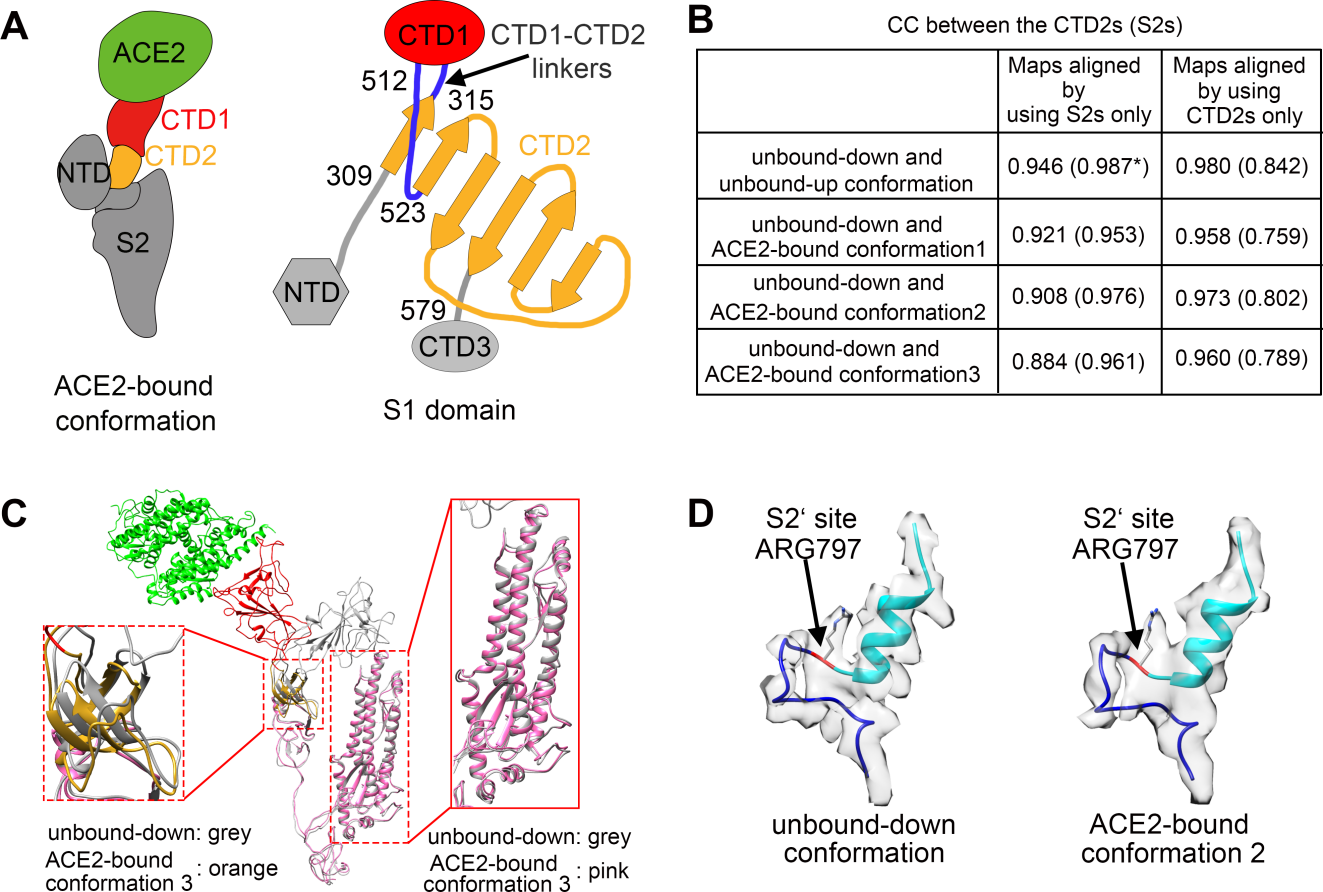
Structural comparisons of the ACE2-bound and ACE2-free SARS-CoV spikes. **(A)** Schematic and topology diagrams showing the domain organization. NTD, CTD3 and S2 are colored grey, CTD1 is colored red, CTD2 is colored orange, CTD1-CTD2 linkers are colored blue. **(B)** Cross-correlation coefficients (CCs) between the CTD2s or the S2s of different conformations. *Values in the parentheses are the CCs between the S2s of different conformations. Density maps were low-pass filtered to 5.5 Å and were compared at a contouring level of 4.0 σ. **(C)** Ribbon-diagram structural comparisons of the ACE2-bound conformation 3 and the unbound-down conformation. ACE2, CTD1, CTD2, CTD3 and S2 of the ACE2-bound conformation 3 are colored green, red, yellow, pink and pink respectively. The unbound-down conformation is colored grey. CTD2 and S2 domain are zoomed in to show the receptor-binding induced hinge motion of CTD2. **(D)** EM densities and corresponding atomic models represented in ribbon diagrams around the S2’ protease cleavage site: unbound-down conformation (left) and ACE2-bound conformation (right). The S2’ site is colored red and position of the S2’ site is indicated with black arrows. The fusion peptide is colored cyan. The “C” shape loop covering the S2’ site is colored blue.

Further comparisons were performed for CTD2, which is located underneath CTD1. CTD2 has close contact with the S2 stem region and is connected to CTD1 through two anti-parallel short hinge linkers (residues 315–322 and residues 512–523) (Fig 2A). Cross-correlation coefficients (CCs) between the CTD2s were calculated with the EM maps aligned using the S2 region or the CTD2 region (Fig 2B). The CCs (average CC between the CTD2s: 0.96) calculated with the maps aligned using CTD2 were much higher than those (average CC between the CTD2s:0.92) calculated with the maps aligned using S2, indicating rigid body movement of CTD2 (Fig 2A and 2B). Model-based structural comparisons showed similar results. The CTD2s of the unbound-up and ACE2-bound conformations exhibited a hinge motion away from the spike axis compared to CTD2 in the unbound-down conformation (Fig 2C). These results indicate that the hinge linker between CTD1 and CTD2 underlies the flexibility of CTD1. Upon binding to the receptor ACE2, CTD2 tended to exhibit a hinge motion away from the S2 stem. The “down” to “up” conformational switch of one CTD1 and its binding to one ACE2 did not induce significant conformational changes in the stem region of the prefusion S glycoprotein.

Exposure of the S2’ cleavage site is assumed to occur after receptor binding [8]. We examined the S2’ cleavage site in different conformational states of the S glycoprotein. The S2’ cleavage site located in a surface pocket of the stem around Arg797 and covered by a “C”-shaped loop (residues 787–796) is inaccessible in both the unbound-down and the ACE2-bound SARS-CoV S glycoprotein structures (Fig 2D). These results indicated that neither the “down” to “up” conformational change of one CTD1 and its binding to one ACE receptor nor a decrease in the pH drive exposure of the S2’ cleavage site.

### Structures of the S2 trimer in a postfusion state and the dissociated S1-ACE2 complex

**Fig 3:**
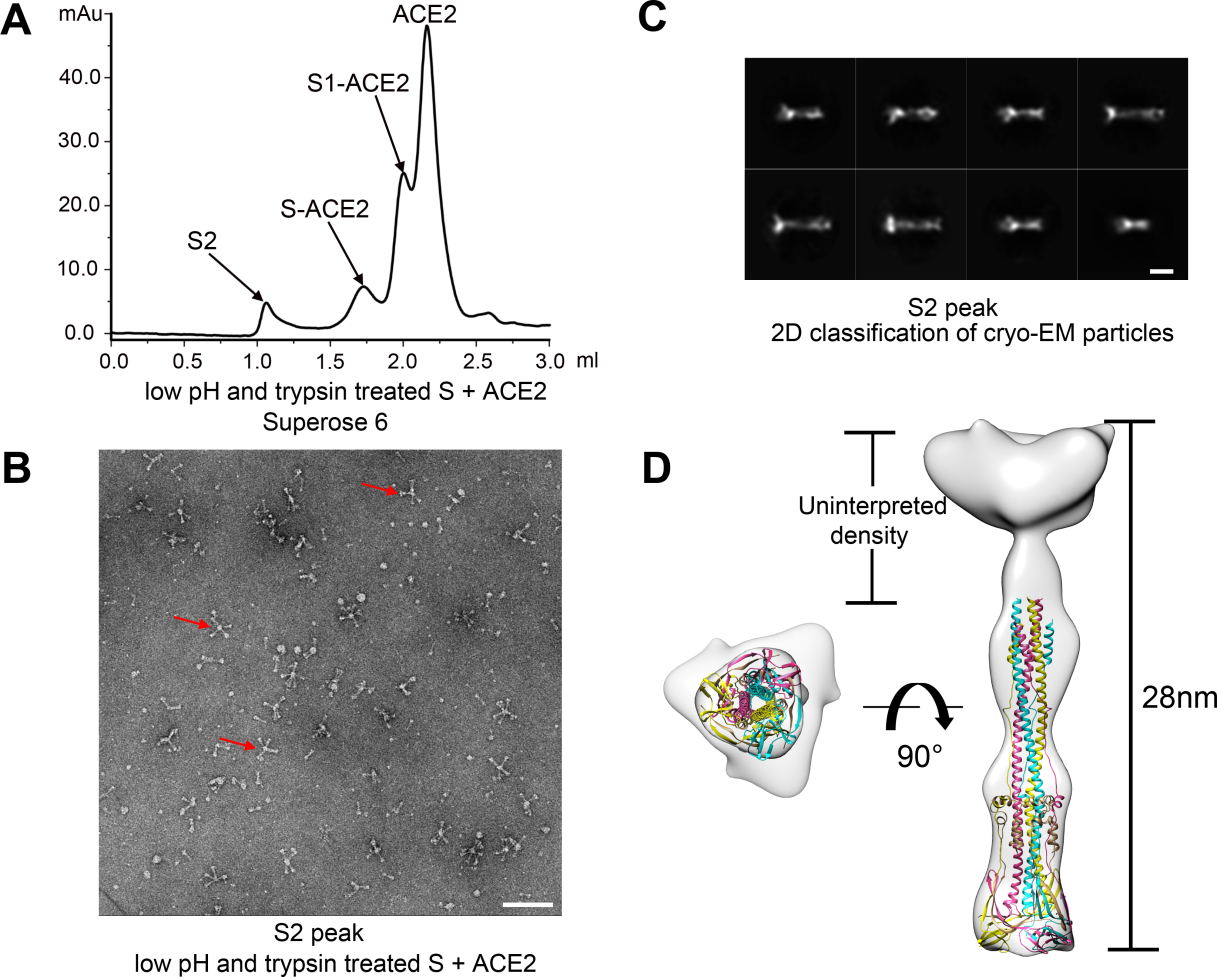
Structure of the post-fusion SARS-CoV S2. **(A)** A size-exclusion chromatography elution profile of the low pH and trypsin treated S and ACE2 mixture. **(B)** Negative staining analysis of the S2 peak. Red arrows indicate rosette-shape structures formed by the post-fusion S2 trimers. Scale bar: 50 nm. **(C)** 2D class averaged cryo-EM images of the SARS-CoV S2 rosette. Scale bar: 10 nm. **(D)** 3D density map of the SARS-CoV S2 in post-fusion state with a fitted SARS-CoV S2 homology model. The three protomers are colored pink, yellow and cyan, respectively. Left: bottom view; right: side view.

Rosette-shaped particles that did not belong to any of the prefusion S glycoprotein states were observed in the EM micrographs of a size-exclusion chromatography elution peak before the S-ACE2 complex peak (Fig 3A and 3B), and 2D and 3D cryo-EM image analysis of the selected particles generated a dumbbell-shaped density map for the petals of the rosette (Fig 3C and 3D). The shape and size of the density map were consistent with the recently determined postfusion MHV S2 trimer structure [18]. Based on the postfusion MHV S2 trimer model, a homologous model of the SARS-CoV S2 trimer in the postfusion state was produced using SWISS-MODEL (S9A and S9B Fig). Fitting of the SARS-CoV postfusion S2 trimer model onto the EM map showed good agreement (Fig 3C). The uninterpreted density at one distal end of the dumbbell-shaped density map should be the exposed fusion peptide that is disordered and mediates the aggregation of the postfusion S2 trimers into the rosette-shaped particles. The S2’ cleavage site is not resolved and should be completely exposed based on the postfusion S2 structure model (S9C Fig).

**Fig 4:**
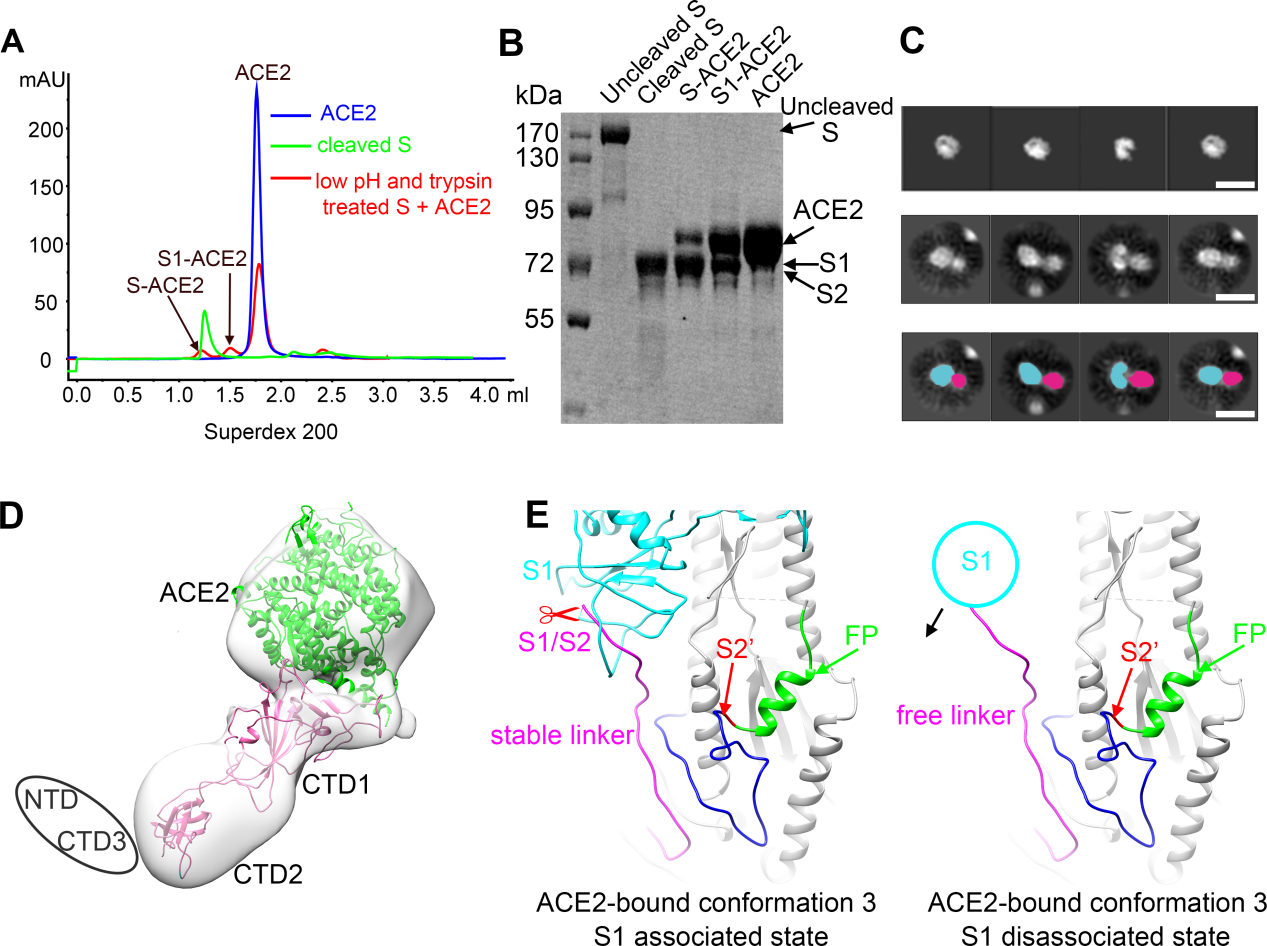
Structure of the disassociated S1-ACE2 complex. **(A)** Size-exclusion chromatography profiles of ACE2 alone (blue), cleaved S (green) and the low pH and trypsin treated S and ACE2 mixture. **(B)** SDS-page analysis of the uncleaved S, cleaved S, S-ACE2, S1-ACE2 and ACE2 peak fractions in “A”. **(C)** 2D analysis of the S1-ACE2 peak in “A”. Upper: 2D projections of the ACE2 density map calculated from the atomic model (PDB ID: 2ajf). Middle: 2D class averaged images of the particles from the S1-ACE2 peak in “A”. Bottom: components of the complex marked in the 2D class averaged images. ACE2 and S1 densities are marked cyan and pink, respectively. **(D)** A 3D density map calculated from the particles of the S1-ACE2 peak. The CTD1, CTD2 (pink) and ACE2 (green) are fitted into the density map as a rigid body. The flexible NTD and CTD3 are not visible and are represented as an ellipse. **(E)** Ribbon diagrams showing the linker downstream the S1/S2 cleavage site and the S2’ cleavage site of ACE2-bound conformation 3 in S1 associated state (left) and S1 disassociated state (right), respectively. The S2’ cleavage site is colored red and indicated by a red arrow. The fusion peptide down-stream of the S2’ site is colored green and the “C” shape loop up-stream the S2’ site is colored blue. The S1 subunit and the linker down-stream the S1/S2 cleavage site of the adjacent protomer is colored cyan and pink, respectively. The black arrow indicates the flexibility of the linker after the disassociation of the S1 subunit.

When preparing the trypsin-cleaved and low-pH-treated S and ACE2 complex, an additional small peak in the middle of the elution profile was observed (Fig 4A). The elution position of the peak indicated a protein or protein complex with a molecular weight of approximately 150 kDa. Subsequent biochemical and cryo-EM analyses of the peak showed a complex of one S1 subunit monomer and one ACE2 molecule (Fig 4B–4D, S10 Fig), suggesting that binding of ACE2 to the S glycoprotein could trigger the dissociation of one S1 from the trimer together with ACE2. Analysis of the S trimer structure in the prefusion state showed that the “C”-shaped loop (residues 787–796) covering the S2’ cleavage site was clipped by the linker downstream of the S1/S2 cleavage site of an adjacent protomer (Fig 4E). Disassociation of one S1-ACE2 complex from the spike would release the clipping linker and could induce conformational changes of the “C”-shaped loop, exposing the S2’ site for protease cleavage and promoting the formation of a postfusion S2 trimer (Fig 4E). Of note, we observed a similar size-exclusion chromatography profile and rosette-shaped particles in the EM image of the complex consisting of ACE2 and the trypsin-cleaved S glycoprotein without low-pH treatment (S11 Fig). This finding indicates that low-pH treatment of the cleaved S glycoprotein is not required for the disassociation of S1-ACE2 or for the formation of rosette-shaped particles of the postfusion S2 trimer. We also incubated the S glycoprotein with ACE2 first and then used low-pH buffer and trypsin to treat the sample. Size-exclusion chromatography analysis of the sample showed a similar elution profile with four peaks consisting of S2, S-ACE2, S1-ACE2 and excess ACE2, respectively (S12 Fig).

## Discussion

Proteolysis is key to coronavirus entry. Unlike the S glycoproteins of MERS-CoV and MHV, the SARS-CoV S glycoprotein is not cleaved at the S1/S2 site during virus packaging in cells and, hence, remains uncleaved on mature virions [22, 23]. However, cleavage of the precursor S protein at the S1/S2 cleavage site by extracellular or endosome proteases is required for a functional SARS-CoV S glycoprotein [24]. Previous studies showed that blockage of the pH decrease in endosomes slightly reduced SARS-CoV infection, which indicated that low pH is not an essential factor for virus entry [25, 26]. Here, our results showed that the prefusion architecture, either in the unbound or ACE2-bound state, was retained in the cleaved S glycoprotein, and the conformational heterogeneity of CTD1s still existed. Similar results were also obtained for the cleaved with/without low-pH treatment S glycoproteins. Therefore, although cleavage at the S1/S2 site is required for a functional S glycoprotein, it does not significantly affect the overall architecture and conformation heterogeneity. However, we did observe significant differences between the mutant and wild-type cleaved S glycoproteins in binding ACE2, indicating that the S1/S2 cleavage site might affect CTD1 receptor binding, although the mechanism is still under investigation.

Receptor binding plays critical roles in facilitating virus-cell attachment and in determining tissue and host tropism [4]. Our complex structures confirmed that the conformational switch of CTD1 from the “down” to “up” position is a prerequisite for receptor binding. Structural comparisons showed that binding of the receptor further opens CTD1. In addition, observations of the S1-ACE2 complex and the postfusion S2 assembly in the receptor-present sample suggested that receptor binding can open one CTD1 and trigger the release of all the S1 subunits from the spike. We did not observe S glycoprotein particles with two remaining S1 subunits in any sample, which suggests that disassociation of one S1-ACE2 from the S trimer could cause sequential disassociation of the S1 subunits from the spike trimer. A similar disassociation of the S1 subunits from the spike was also observed for MERS-CoV, even without binding the receptor [7, 16]. Although the virus uses a similar CTD1 “up” mechanism for receptor binding, the MERS-CoV S glycoprotein, which can have all three CTD1s of a trimer spike in the “up” conformation, is significantly different from the SARS-CoV S glycoprotein, in which only one “up” CTD1 was observed. In addition, disassociated trimeric S1 particles were observed for MERS-CoV [16], suggesting simultaneous disassociation of all three S1 subunits from the MERS-CoV S glycoprotein. Structural modeling showed that sufficient space exists for the other two CTD1s in the “down” position to point “up” and to bind with ACE2 for most of the S-ACE2 complexes (S13 Fig). However, we did not observe more than one ACE2 or more than one “up” CTD1 in one spike. We speculate that a conformational switch producing more than one “up” CTD1 may destabilize the cleaved S glycoprotein and trigger immediate disassociation of the S1 subunits and the S1-ACE2 complex. Additionally, disassociation of the SARS-CoV S1 subunit should be receptor binding-dependent, since no free S1 was observed when ACE2 was missing.

**Fig 5:**
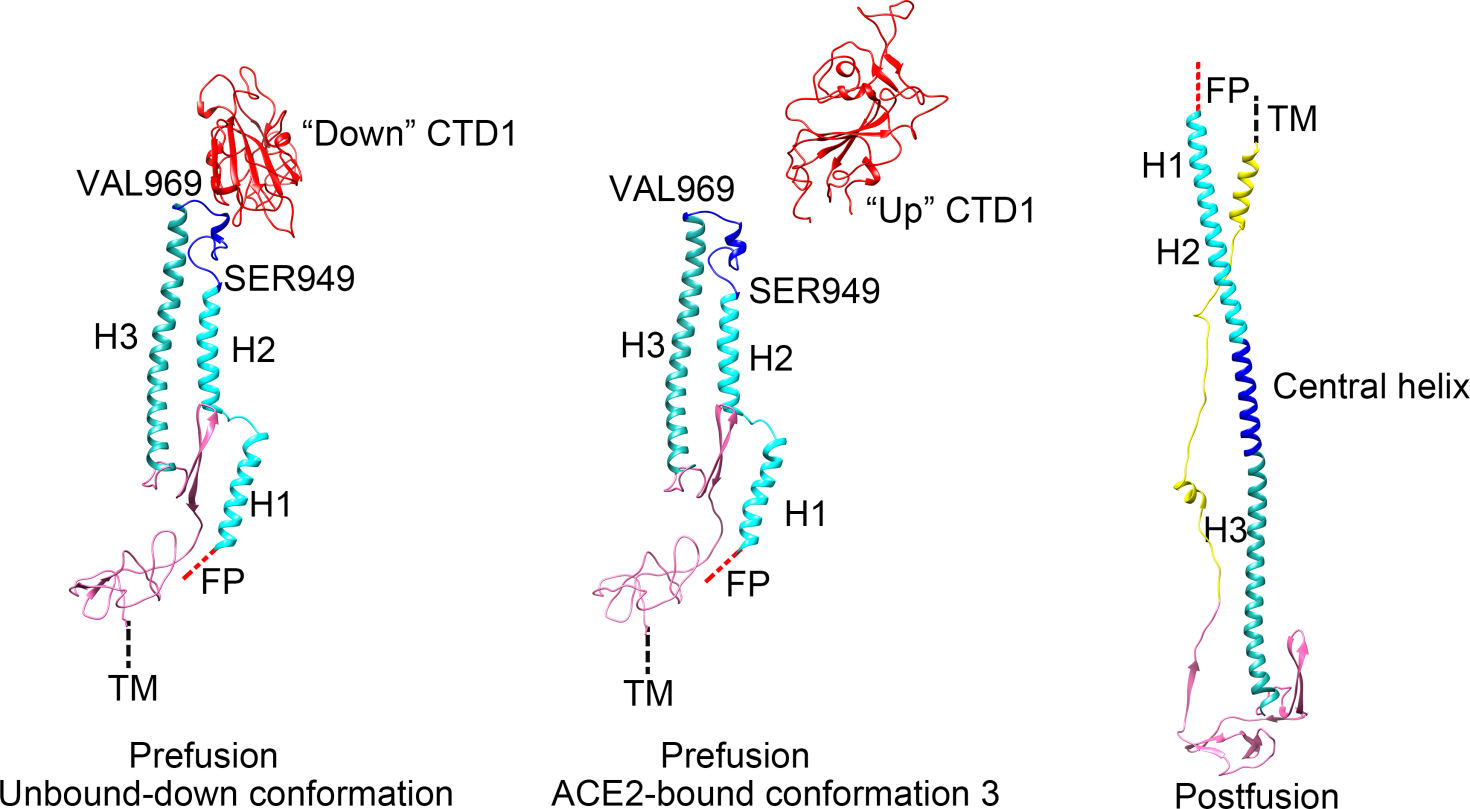
The pre- to post-fusion structural transition of the S2 subunit. Left: ribbon diagrams showing the prefusion SARS-CoV S2 structure in which the “down” CTD1 has direct contacts with the helix linker 2 (blue). H1: helix 1. H2: helix 2. H3: helix 3. H1, H2 and helix linker 1: cyan. Helix linker 2: blue. H3: dark green. CTD1: red. Connecting domain: pink. Middle: ribbon diagrams showing the S2 region of the S-ACE2 complex in which the “up” CTD1 has no direct contacts with the Helix linker 2. Right: ribbon diagram of the postfusion SARS-CoV S2. H1, H2, H3 and the linkers transit to form the long central helix. HR2: yellow.

The prefusion S2 subunits assemble to form a nine-helix bundle in the central core region. The three helix fragments (H1, H2 and H3) of each S2 subunit are connected through two short linkers (helix linker 1: residues 921 to 927; helix linker 2: residues 949 to 969) (Fig 5). The postfusion S2 subunits are a six-helix bundle with a 160 Å-long central helix core. The pre- to postfusion transition of the S2 subunits requires a 180° flip of prefusion helix fragments H1 (residues 902–920) and H2 (residues 928–948) that fuse with helix fragment H3 (residues 970–1015) to form the postfusion central helix core. Helix linker 2 is critical for the formation of the postfusion helix core, as has been shown in a previous study, which indicated that substitution of the residues in this region with prolines retains the S glycoprotein in the prefusion conformation and prevents conformational rearrangement [7]. The “down” CTD1s are located immediately above the S2 subunits and have direct contact with helix linker 2 (Fig 5, left). The direct contact should stabilize the prefusion S2 subunit and prevent it from transitioning into the postfusion state. Opening of CTD1, especially by binding the receptor, would remove the steric restraints on helix linker 2, triggering the release of the S1 subunits and probably simultaneously allowing the extension of prefusion S2 helixes to form the postfusion S2 long helix bundle (Fig 5).

**Fig 6:**
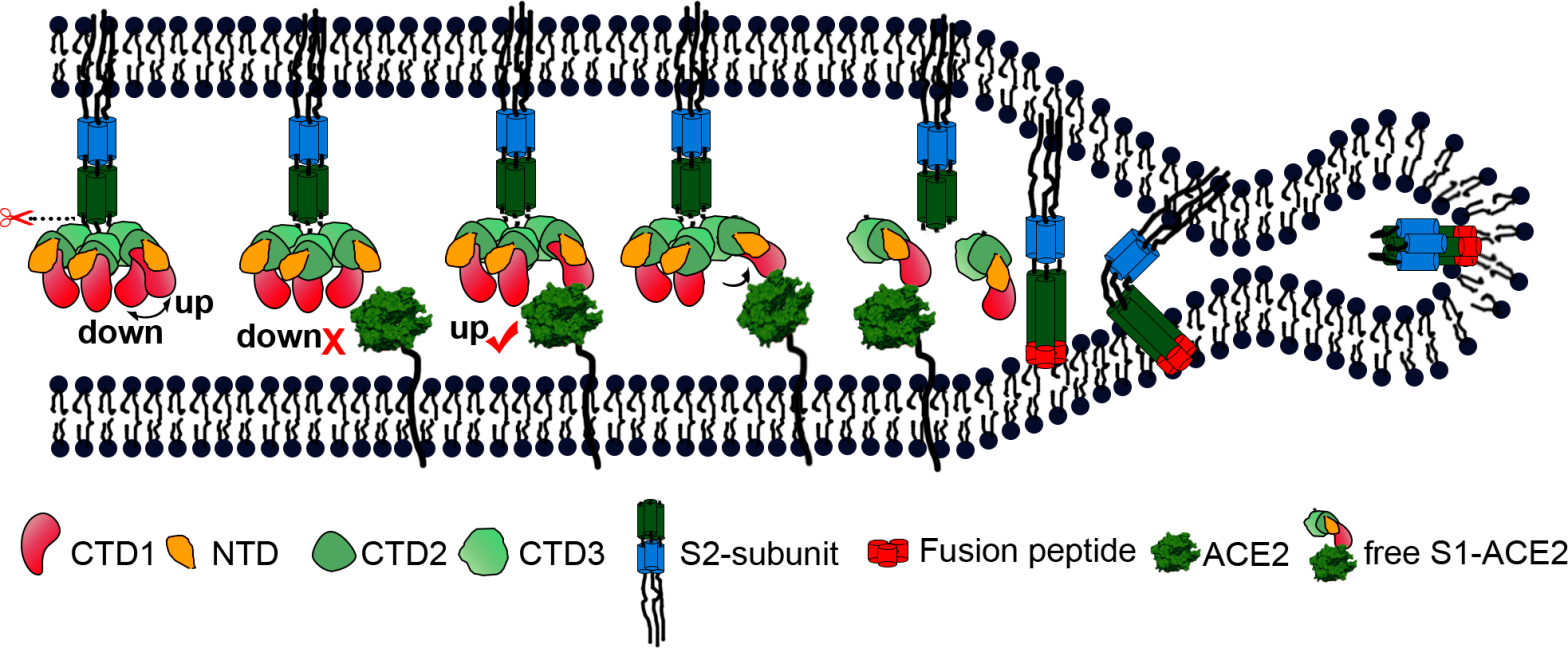
A cartoon representation showing the pre- to post-fusion transition of the SARS-CoV S glycoprotein. The “down” to “up” transition of the receptor-binding domain (CTD1) allows receptor binding. The binding to ACE2 opens up CTD1 and CTD2, promotes the disassociation of the S1-ACE2 complex from the S1/S2 cleaved S glycoprotein, induces the pre- to post-fusion transition of the S2 subunit, and initiates the membrane fusion.

These new data allow us to reorganize and optimize the current model for SARS-CoV entry (Fig 6). However, some details are still missing, including the exact function of the S2’ cleavage site and the time point when this site is cleaved, which may be illustrated by further investigation.

## Materials and methods

### Gene cloning and protein purification

A human codon-optimized gene coding the SARS-CoV S glycoprotein ectodomain (NCBI Accession NP_828851.1) residues 1–1195 fused with a C-terminal strep tag for purification was cloned and inserted into a pFastBac-Dual vector (Invitrogen). The recombinant SARS-CoV S glycoprotein ectodomain was generated in Hi5 insect cells, purified by Strep-Tactin Sepharose (IBA GmbH) and concentrated to less than 100 μl for gel-filtration chromatography using an increase Superdex 200 column (GE Healthcare) pre-equilibrated with HBS buffer (10 mM HEPES at pH 7.2, 150 mM NaCl).

Human ACE2 extracellular domain (residues 19–615) with an N-terminal gp67 signal peptide for secretion and a C-terminal 6 × his tag for purification was inserted into a pFastBac-Duel vector (Invitrogen). The construct was transformed into bacterial DH10Bac component cells using Cellfectin II Reagent (Invitrogen). After 5 days of incubation at 27°C, the low-titer viruses were harvested and amplified to P2 and then used to infect Sf9 cells. The cell culture was collected by centrifugation at 48 hours post infection. The supernatant was concentrated, loaded into nickel (Ni)-charged resin (GE Healthcare), eluted with 0.5 M imidazole in Buffer A (50 mM Tris, pH 8.8, 40 mM NaCl) and further purified using an increase Superdex 200 high-performance column (GE Healthcare) pre-equilibrated with Buffer A. Fractions containing ACE2 were collected, applied directly to a pre-equilibrated Resource Q column (GE Healthcare), and then eluted with a 0.05–1 M NaCl gradient in 40 mM Tris buffer (pH 8.8). Fractions containing ACE2 were then purified using an increase Superdex 200 column pre-equilibrated with HBS buffer.

### Trypsin and low-pH treatments of the SARS-CoV S glycoprotein

L-(tosylamido-2-phenyl) ethyl chloromethyl ketone (TPCK)-treated trypsin was added to the purified SARS-CoV S glycoprotein at a mass ratio of 1:100 in 50 mM Tris-HCl at pH 8.0 with 20 mM CaCl_2_. The mixture was incubated at 18°C for more than 16 hours and then purified by gel-filtration chromatography (Superose 6 5/150) running in HBS to remove trypsin. Low-pH treatment of the sample was performed by adding 1/10 volume of 1 M sodium citrate stock at pH 5.6, followed by incubation at room temperature for 2 hours. To prepare the complex of the cleaved SARS-CoV S glycoprotein with its receptor, ACE2, the cleaved S protein was mixed with ACE2 at a molar ratio of 1:3, and then the mixture was incubated at 4°C overnight. The mixture was purified and verified by gel-filtration chromatography using an increase Superose 6 5/150 column (GE Healthcare) pre-equilibrated with HBS buffer.

### Receptor, low-pH and trypsin treatments of the SARS-CoV S glycoprotein

Purified S glycoprotein and ACE2 were mixed at a molar ratio of more than 1:3 and were incubated at 4°C overnight. Then, 1/10 volume of 1 M sodium citrate stock at pH 5.6 was added to the mixture, followed by incubation at room temperature for 2 hours. Next, the mixture was treated with trypsin at a mass ratio of 1:100 in 50 mM Tris-HCl at pH 8.0 with 20 mM CaCl_2_ at 18°C, for more than 16 hours. The mixture was purified by filtration chromatography using an increase Superose 6 5/150 column (GE Healthcare) pre-equilibrated with HBS buffer. For native page gel analysis, purified S glycoprotein and trypsin-cleaved S glycoprotein were with mixed with 5x native loading buffer (30% glycerol, 100 mM Tris-HCl, pH 8.0, 1% bromophenol blue). The native page gel consisted of an acrylamide gradient ranging from 4% to 15%.

### Immunoblotting

Uncleaved S glycoprotein, cleaved S glycoprotein, S1-ACE2 and ACE2 were prepared as previously described in this paper. The samples were mixed with loading buffer and boiled at 100°C for 10 minutes. The samples were separated by electrophoresis on a 12% (w/v) sodium dodecyl sulfate-polyacrylamide (SDS-page) gel and were then transferred to a nitrocellulose membrane. The membrane was blocked for 1 hour at room temperature with a non-fat milk buffer and then incubated with mouse monoclonal anti-strep tag antibodies (HX1816, Huaxingbio Biotechnology, Beijing, China), mouse monoclonal anti-6x his tag antibodies (HX1804, Huaxingbio Biotechnology, Beijing, China), or mouse anti-CTD1 serum for 1 hour at room temperature. Then, the membrane was incubated with goat-anti-mouse IgG (HX2032, Huaxingbio Biotechnology, Beijing, China) for 45 minutes at room temperature. The blots were visualized by using ECL chemiluminescence reagents (HX1868, Huaxingbio Biotechnology, Beijing, China). An Amersham Image 600 device (GE Healthcare) was used to analyze the results. Mouse monoclonal anti-strep tag antibodies and mouse monoclonal anti-6x His tag antibodies were diluted at 1:5000, and mouse anti-CTD1 serum (provided by Panpan Zhou from Linqi Zhang’s lab at Tsinghua University) was diluted at 1:1000.

### Negative-staining electron microscopy

For negative-staining sample preparation, 3 μl aliquots of samples at a concentration of ~0.02 mg/ml were applied onto a glow-discharged grid with a continuous carbon layer (Beijing Xinxing Braim Technology Co., Ltd.). Excess sample was removed using filter paper after 1 minute of incubation on the carbon grid. The grid was immediately washed twice and incubated with 3 μl of 1% uranyl acetate (UA) solution for an additional minute. The grid was then blotted with filter paper to remove UA, air-dried at room temperature, and examined under an FEI Tecnai Spirit electron microscope operating at an acceleration voltage of 120 keV. Images were collected using a Gatan 895 4 k × 4 k CCD camera at a nominal magnification of 49,000× with a pixel size of 0.227 nm.

### Cryo-EM

For cryo-EM sample preparation, 3 μl aliquots of samples at a concentration of ~0.2 mg/ml were applied to glow-discharged holey carbon grids (Quantifoil, Cu 200 mesh, R1.2/1.3) or grids with a layer of continuous ultrathin carbon film (Ted Pella, Inc.). The grids were blotted and then were plunged into liquid ethane using an FEI Vitrobot Mark IV. The concentration of the S2 rosette sample was low. Aliquots (3 μl) of the S2 rosette sample were applied to the grids, and excess sample was removed using filter paper after 30 s. This procedure was repeated two times. When the sample was applied to the grid for the third time, the grids were blotted and then submerged in liquid ethane as described above.

Images of the S2 rosette, trypsin-cleaved S glycoprotein, trypsin-cleaved and low-pH-treated S glycoprotein, and the complex of ACE2 and trypsin-cleaved S glycoprotein (S11A Fig, S-ACE2 peak) were collected in frame stacks using an FEI Tecnai Arctica electron microscope operating at an acceleration voltage of 200 keV and equipped with a Falcon II direct electron detector. Images were recorded at a defocus range of -1.5 μm to -4 μm with a pixel size of 1.27 Å. The exposure time was 1.2 s, with a total exposure dose of ~60 electrons per Å^2^ over 19 frames.

Images of the ACE2 and cleaved and low-pH-treated S glycoprotein complex (Fig 4A, S-ACE2 peak) and the disassociated ACE2 and S1 complex (Fig 4A, S1-ACE2 peak) were collected using an FEI Titan Krios electron microscope operating at an acceleration voltage of 300 keV and equipped with a Gatan K2 Summit direct electron detector. Images were recorded at a defocus range of -1.5 μm to -4 μm with a pixel size of 0.66 Å. Each image was dose-fractionated into 32 movie frames at a dose rate of 8.2 counts per physical pixel per second, with a total exposure time of 8 s and a frame exposure time of 0.25 s, resulting in a total dose of ~50 electrons per Å^2^. Data collection and image processing statistics are listed in S1 and S2 Tables.

### Image processing

Movie frames of the K2 image stacks collected for the ACE2 and trypsin-cleaved and low-pH-treated S complexes (Fig 4A, S-ACE2 peak) were 2 × 2 down sampled, resulting in a pixel size of 1.32 Å, and then were aligned using the MOTIONCORR2 program [27] before further processing. The CTF parameters were determined with the Gctf program [28]. Particles were automatically selected using the Gautomatch program [29]. Particles were visually inspected to remove false positives. 2D and 3D classifications and refinements were performed using RELION 1.4 [30]. The density map of SARS-CoV S glycoprotein in the prefusion state (EMDB ID: EMD-6732) was low-pass filtered to 40 Å and was used as an initial model for 3D analysis. In total, 1,033,788 particles selected from 2,813 micrographs were subjected to several rounds of 2D classification, and 688,289 selected particles were then subjected to 3D classification, yielding three classes of S glycoproteins with ACE2 bound and two classes of S glycoproteins free of ACE2. One of the ACE2-free classes was three-fold symmetrical, with all three CTD1s in the “down” conformation (unbound-down), and the other ACE2-free class was asymmetrical, with one CTD1 in the “up” conformation and two CTD1s in the “down” conformation (unbound-up). The particles from the symmetrical unbound-down class were sorted according to their *LogLikeliContribution* values, and particles with higher values were selected and subjected to 3D auto-refinement with C3 symmetry imposed, yielding a density map of 3.6 Å. Particles from the unbound-up class were further selected through 3D classification with a mask excluding the flexible “up” CTD1. Selected particles were used for auto-refinement and reconstruction, which resulted in a final density map of 3.9 Å. Similar procedures were also used for the reconstructions of the ACE2-bound particles, yielding three ACE2-bound reconstructions with resolutions of 5.4 Å, 4.2 Å and 4.5 Å.

Movie frames of the K2 dataset of the ACE2-S1 complex (Fig 4A, S1-ACE2 peak) were aligned, and the CTF parameters were determined using a similar strategy to that used for the ACE2 and trypsin-cleaved or low-pH-treated S glycoprotein complexes. A total of 241,551 particles were automatically selected using the Gautomacth program. Then, 2D and 3D classifications and refinements were performed using RELION 1.4. An initial model was generated by *e2initialmodel*.*py* using typical 2D class averages. To validate the accuracy of the reconstruction, another run of 3D autorefine was performed using the same particle dataset but with a different initial model of ACE2. The ACE2 atomic model (PDB ID: 2ajf) was converted into a density map using the python script *e2pdb2mrc*.*py* [31]. Both initial density maps were low-pass filtered to 40 Å before being used for 3D analysis. The two reconstructions had a CC value of 0.88 (at a contouring level of 3 σ), and the consistency of the two reconstructions indicated that the reconstruction was reliable. The 2D projections of ACE2 were generated with *e2project*.*py* [31].

The Falcon II datasets were processed in a similar manner to that used for the K2 dataset of the ACE2 and trypsin-cleaved or low-pH-treated S glycoprotein complexes. For the ACE2 and trypsin-cleaved S glycoprotein complex (S11A Fig, S-ACE2 peak), a total of 324,514 particles were automatically selected using the Gautomacth program and then were 2 × 2 down sampled before further processing. The 2D classification yielded 224,052 selected particles, and 3D classification yielded five conformational states corresponding to ACE2-bound S glycoprotein conformation 1, ACE2-bound S glycoprotein conformation 2, ACE2-bound S glycoprotein conformation 3, unbound-up S glycoprotein and unbound-down S glycoprotein. Then, 3D auto-refinements were conducted for particles of each class, yielding a density map of 19.7 Å for the ACE2-bound S glycoprotein conformation 1, a density map of 9.0 Å for the ACE2-bound S glycoprotein conformation 2, a density map of 18.5 Å for the ACE2-bound S glycoprotein conformation 3, a density map of 9.0 Å for the unbound-up S glycoprotein and a density map of 8.3 Å for the unbound-down S glycoprotein subset. For the Falcon II dataset of the S2-rosette, a total of 34,149 particles were subjected to several rounds of 2D and 3D classification. An initial model was generated by *e2initialmodel*.*py* using typical 2D class averages. In total, 7,636 selected particles were used for auto-refinement, yielding a density map of 30.5 Å. For the Falcon II dataset of the trypsin-cleaved S glycoprotein and the trypsin-cleaved then low-pH-treated S glycoprotein, the density map of SARS-CoV S in the prefusion state (EMDB ID: EMD-6732) was low-pass filtered to 40 Å and used as an initial model for 3D analysis. After several rounds of 2D and 3D classification, a density map of 6.8 Å for the trypsin-cleaved S glycoprotein and a density map of 6.7 Å for the trypsin-cleaved then low-pH-treated S glycoprotein were generated.

Map sharpening and B-factor application were performed using the *phenix*.*auto_sharpen* [32] and *relion_postprocess* programs. The reported resolutions are based on the gold-standard Fourier shell correlation (FSC) 0.143 criterion. Local resolution variations were estimated using ResMap [33].

### Structure modeling

Structure modeling statistics of the S-ACE2 complex are listed in S3 Table.

Atomic models were built for three density maps of the ACE2-S complex, the 3.9 Å density map of the unbound-up S glycoprotein and the 3.6 Å density map of the unbound-down S glycoprotein.

For the model building of the 3.6 Å unbound-down S glycoprotein density map, an atomic model of the prefusion S glycoprotein (PDB ID: 5xlr) was used as a reference [34]. The NTDs that were missing in the initial model were built with the atomic model of the NTD crystal structure (PDB ID: 5x4s). The model was refined by using ROSETTA [35] and PHENIX with secondary structure restraints and geometry restraints [32]. Manual adjustments of the model were performed in COOT [36].

For the unbound-up S model, the unbound-down S model excluding the “up” CTD1 was fitted into the 3.9 Å density map as a rigid body. The initial “up” CTD1 was built by fitting the crystal structure of CTD1 to the EM density. Fitting of the atomic models to the cryo-EM densities was performed by maximizing the density value around the fitted atoms [37]. The separately fitted models were combined to yield a complete model. The model was then refined using PHENIX real-space refinement with secondary structure restraints and geometry restraints and adjusted in COOT. CTD1 was defined as a rigid body during the refinement. The models of the ACE2-S complex were generated in a manner similar to that used for the unbound-up S glycoprotein model. The crystal structure of the CTD1-S complex (PDB ID: 2ajf) was used to build the initial model by fitting the structure as a rigid body into the EM density. Molprobity was used to evaluate the final refined models [38].

CCs between the aligned maps were calculated using the UCSF Chimera “measure correlation” command. The CC was calculated using the following formula: CC = <**u**, **v**>/|**u**||**v**|, where vector **u** contains the values of the first map and vector **v** contains the values of the second map. The calculation includes only the grid points in the first map with values above the stated contour level.

To calculate the “up” angles of the CTD1s, the horizontal plane of the S glycoprotein perpendicular to the 3-fold axis and the long axis of the “up” CTD1 were generated using the UCSF Chimera “define” command, and then the angle between the axis and the plane was calculated using the UCSF Chimera “angle” command. The homologous model of the SARS-CoV S glycoprotein in postfusion state was calculated using the MHV S glycoprotein in the postfusion state (PDB ID: 6b3o) as reference model with SWISS-MODEL [39]. All figures were generated with UCSF Chimera [34].

### Accession numbers

The coordinates and EM maps have been deposited into the Protein Data Bank and the EM Data Bank with the accession numbers: 6ACG, 6ACJ, 6ACK, 6ACD, 6ACC, EMD-9591, EMD-9593, EMD-9594, EMD-9589, EMD-9588, EMD-9598, EMD-9597, EMD-9595, EMD-9596, EMD-9585, EMD-9586, EMD-9587, EMD-9584 and EMD-9583.

## Supporting information

S1 Fig Analysis of the mutant R667A SARS-CoV S glycoprotein. **(A)** Schematic diagrams showing the domain organization of the SARS-CoV S glycoprotein. NTD: N-terminal domain, CTD1: C-terminal domain 1 (receptor binding domain, RBD), CTD2: C-terminal domain2 (subdomain 1, SD1), CTD3: C-terminal domain (subdomain 2, SD2), FP: fusion peptide, HR1: heptad repeat 1, HR2: heptad repeat 2. S1/S2 and S2’ protease cleavage sites are indicated with black arrows. **(B)** Representative 2D class averaged images of the ACE2-bound and ACE2-free spikes of the SARS-CoV glycoprotein mutant R667A. Red arrow points to the density of the bound ACE2. Scale bar: 10 nm.
(TIF)

S2 Fig SDS-page, native-page, cryo-EM 2D and 3D analysis of the SARS-CoV S after trypsin cleavage and low pH treatment. **(A, B)** SDS-page and native-page analysis of the cleaved and uncleaved SARS-CoV S glycoprotein. **(C, D)** Representative 2D class averaged images of the cleaved SARS-CoV S glycoprotein at pH 7.2 (C) or pH 5.6 (D). Scale bar: 10 nm. **(E, F)** Cryo-EM 3D reconstruction of the cleaved SARS-CoV S glycoprotein trimer at pH 7.2 (E) or at pH 5.6 (F). The SARS-CoV S glycoprotein atomic model (cyan, PDB ID: 5xlr) is fitted into each 3D density map. Outer surface: left. Central section; right.
(TIF)

S3 Fig Cryo-EM data processing flowchart of the trypsin-cleaved and then low pH treated S and ACE2 complex. See Materials and Methods for details.
(TIF)

S4 Fig Cryo-EM data processing of the trypsin-cleaved and then low pH treated S and ACE2 complex. **(A-B)** A representative raw micrograph (A) and representative 2D class averaged images (B) of the complex. Scale bar in (A): 50 nm. Scale bar in (B): 10 nm. **(C)** Fourier shell correlation (FSC) curves of the 3D reconstructions. ACE2-bound conformation 1: pink, ACE2-bound conformation 2: yellow, ACE2-bound conformation 3: red, unbound-up conformation: cyan, unbound-down conformation: grey. **(D)** Local resolution maps, partial maps, and particle orientation distributions of the 3D reconstructions. From left to right: ACE2-bound conformation 1, ACE2-bound conformation 2, ACE2-bound conformation 3, unbound-up and unbound-down S conformation. Up: local resolution maps of the 3D reconstructions; middle: cryo-EM densities of a selected representative region; bottom: particle orientation distributions of the 3D reconstructions shown around the corresponding EM map.
(TIF)

S5 Fig Atomic models of the ACE2-bound and ACE2-free SARS-CoV spikes in low pH and trypsin treated S and ACE2 complex. From left to right: ribbon diagrams showing the atomic models of the ACE2-bound conformation 1, the ACE2-bound conformation 2, the ACE2-bound conformation 3,unbond-up and unbound-down conformations of the SARS-CoV S glycoprotein after trypsin cleavage and low pH treatment, respectively. ACE2 binding monomer is colored red and the bound ACE2 is colored green. The angle between the long axes of the CTD1 and the horizontal plane is shown at the bottom of each structure.
(TIF)

S6 Fig Structural comparisons of the ACE2-bound and unbound states. From left to right: surface shadowed diagrams showing the top views of the ACE2-bound conformation 1, the ACE2-bound conformation 2, the ACE2-bound conformation 3,unbond-up and unbound-down conformations. The CTD1s are colored pink. The black arrows indicate the “down” CTD1s, the pink arrows indicate the “up” CTD1s. The angle between the long axes of the CTD1 and the horizontal plane is shown at the bottom of each conformation.
(TIF)

S7 Fig Cryo-EM analysis of the trypsin treated S-ACE2 complex at pH 7.2. **(A)** 2D class averaged images of the trypsin treated S-ACE2 complex. Scale bar: 10 nm. **(B)** 3D density maps of the ACE2-bound conformation 1, ACE2-bound conformation 2, ACE2-bound conformation 3, unbound-up, and unbound-down conformations (from left to right). **(C)** Fourier shell correlation (FSC) curves of the 3D reconstructions. ACE2-bound conformation 1: pink, ACE2-bound conformation2: yellow, ACE2-bound conformation 3: red, unbound-up conformation: cyan, unbound-down conformation: grey.
(TIF)

S8 Fig EM densities of the CTD2s. From left to right: unbound-down CTD2 (contouring level: 8 σ), unbound-up CTD2 (contouring level: 8 σ) and ACE2-bound conformation3 CTD2 (contouring level: 8 σ). The C**α** backbone of the CTD2 is shown in each density map.
(TIF)

S9 Fig Homology model of the post-fusion SARS-CoV S2. **(A)** Model of the post-fusion MHV S2. The three protomers are colored pink, yellow and cyan, respectively. **(B)** Model of the SARS-CoV post-fusion S2 obtained through homology modeling using the model in “A”. **(C)** Model of the SARS-CoV post-fusion S2 showing the possible locations of the S2’ cleavage site and the fusion peptide (FP, colored red).
(TIF)

S10 Fig Western blot analysis of the size-exclusion chromatography peak fractions of the low pH and trypsin treated S and ACE2 complex. Samples are related to Fig 4B. The C-terminus of the S2 subunit contains a strep tag. ACE2 has a C-terminal his tag. Anti-CTD1 serum is generated by immunizing the mouse with the CTD1 (residue: 327–516) of the SARS-CoV S glycoprotein. The bands are detected by using anti-strep mono-antibody (**A**), anti-his mono-antibody (**B**) or anti-CTD1 serum (**C**).
(TIF)

S11 Fig Gel filtration and negative staining analysis of the trypsin treated S and ACE2 complex at pH 7.2. **Left:** size-exclusion chromatography elution profile of the trypsin treated S and ACE2 mixture at pH 7.2. Four peaks were observed, similar to that of the low pH treated sample as in Fig 3A. **Right:** negative staining analysis of the peak S2. Red arrows indicate the rosette-shape structures formed by the post-fusion S2 trimers. Zoom-in images are shown below the raw micrograph. Scale bar: 50 nm.
(TIF)

S12 Fig Gel filtration and SDS page analysis of the uncleaved S and ACE2 followed by low pH and trypsin treatment. **Left:**Size-exclusion chromatography elution profile. Four peaks were observed, similar to the profile as in Fig 3A. **Right:** SDS-page analysis of the peak fractions. From left to right: marker, uncleaved S, cleaved S, S-ACE2 peak, S1-ACE2 peak and ACE2 peak.
(TIF)

S13 Fig Structure modeling of three “up” CTD1s binding ACE2s. **(A)** Three conformation 1 “up” CTD1s binding ACE2s. The CTD1s are colored pink. Three ACE2s are colored green. The volume of the steric clashes between two neighboring monomers is 9,406 Å^3^ and is colored yellow. **(B)** Three conformation 2 “up” CTD1s binding three ACE2s. **(C)** Three conformation 3 “up” CTD1s binding three ACE2s. The CTD1s and ACE2 are colored the same as in “A”. No steric clash was observed for structure models in “B” and “C”. Top: side views. Middle: top views. Bottom: the angle between the long axes of the CTD1 and the horizontal plane.
(TIF)

S1 Table 3D Classification statistics of different conformational states of the S-ACE2 complex. (DOCX)

S2 Table Cryo-EM data collection and image processing statistics. (DOCX)

S3 Table Structure modeling statistics of the ACE2-bound and ACE2-free SARS-CoV spikes. (DOCX)
