# Supplementary figures and images for: Cryo-EM structure of the SARS coronavirus spike glycoprotein in complex with its host cell receptor ACE2
Source: PLoS Pathog. 2018 Aug 13;14(8):e1007236. doi: 10.1371/journal.ppat.1007236 (PMC6107290; doi:10.1371/journal.ppat.1007236)

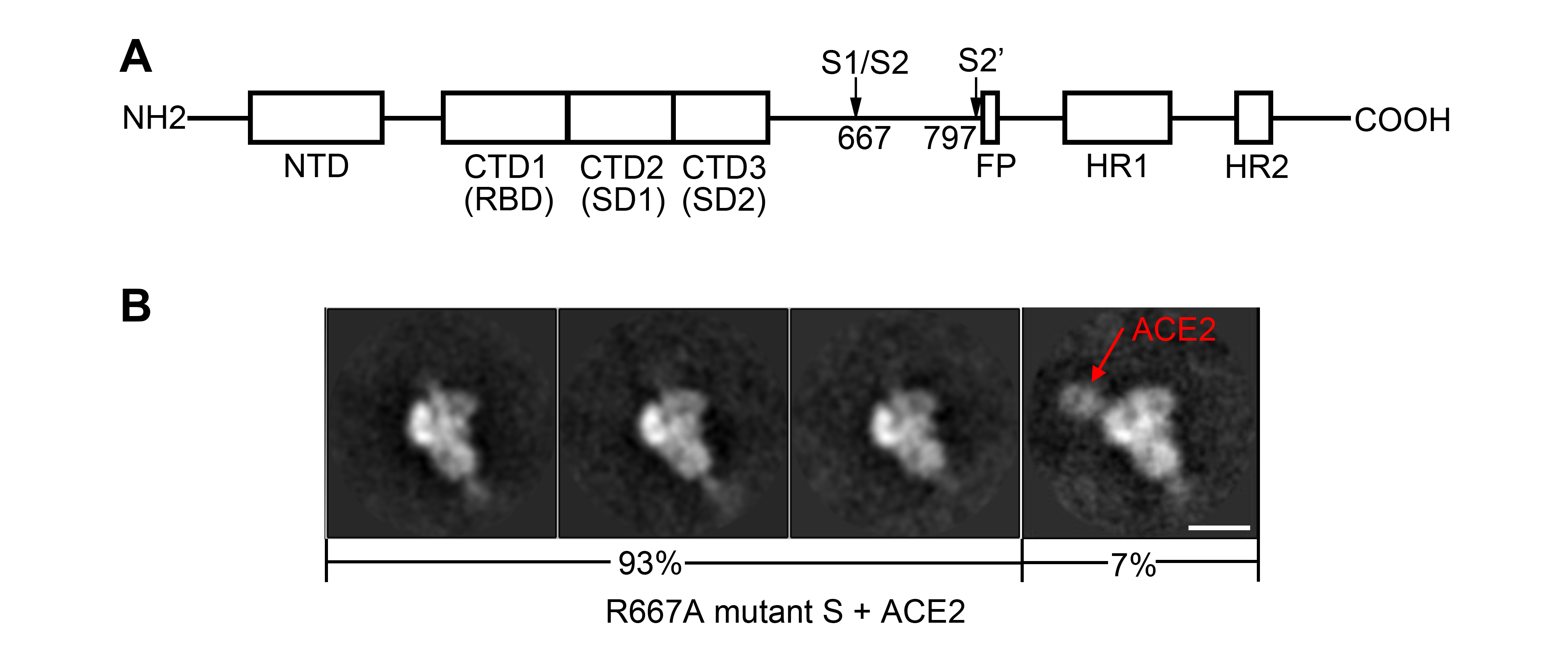

Supplement: S1 Fig — (A) Schematic diagrams showing the domain organization of the SARS-CoV S glycoprotein. NTD: N-terminal domain, CTD1: C-terminal domain 1 (receptor binding domain, RBD), CTD2: C-terminal domain2 (subdomain 1, SD1), CTD3: C-terminal domain (subdomain 2, SD2), FP: fusion peptide, HR1: heptad repeat 1, HR2: heptad repeat 2. S1/S2 and S2’ protease cleavage sites are indicated with black arrows. (B) Representative 2D class averaged images of the ACE2-bound and ACE2-free spikes of the SARS-CoV glycoprotein mutant R667A. Red arrow points to the density of the bound ACE2. Scale bar: 10 nm. (TIF) [file ppat.1007236.s001.tif]

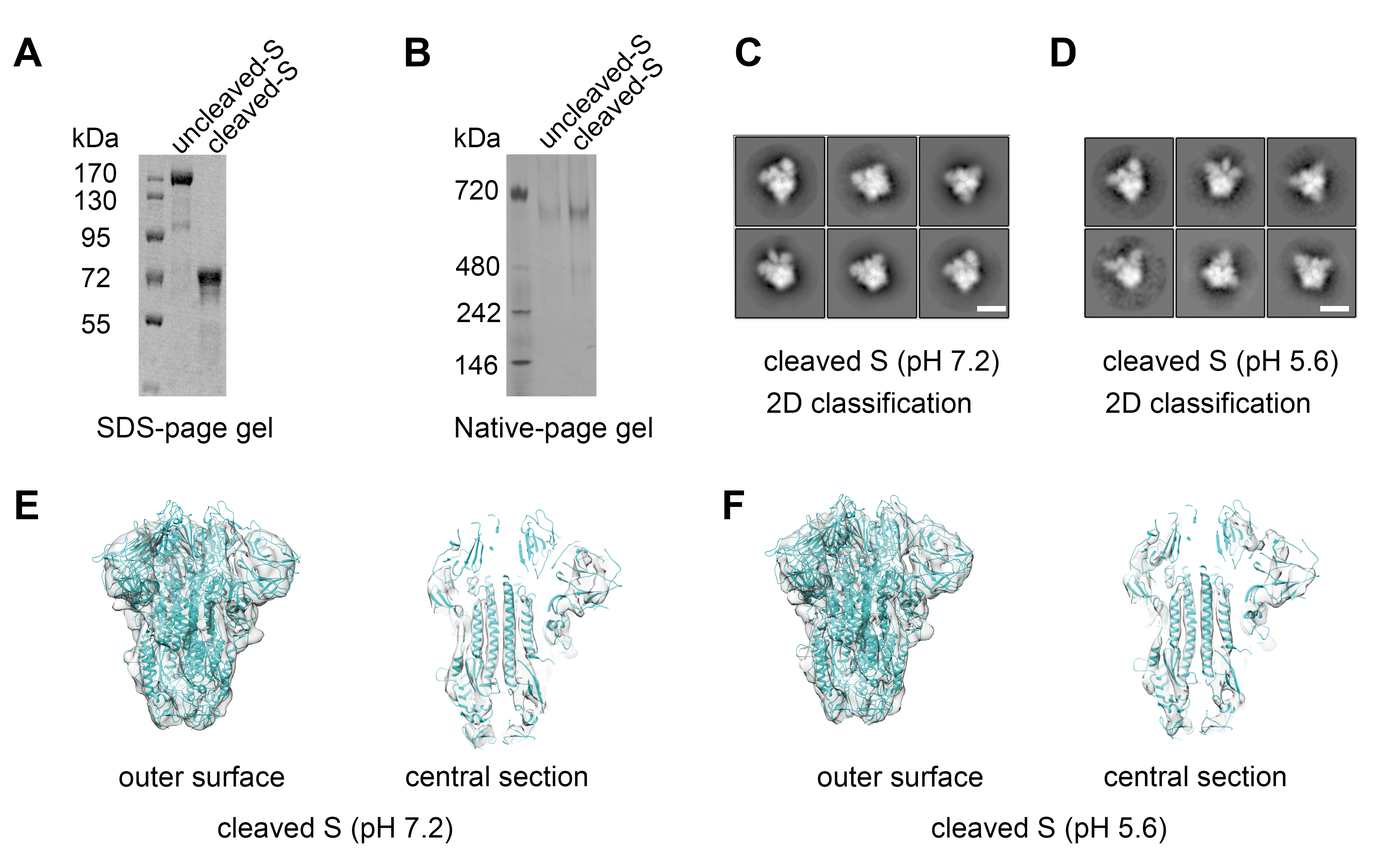

Supplement: S2 Fig — (A, B) SDS-page and native-page analysis of the cleaved and uncleaved SARS-CoV S glycoprotein. (C, D) Representative 2D class averaged images of the cleaved SARS-CoV S glycoprotein at pH 7.2 (C) or pH 5.6 (D). Scale bar: 10 nm. (E, F) Cryo-EM 3D reconstruction of the cleaved SARS-CoV S glycoprotein trimer at pH 7.2 (E) or at pH 5.6 (F). The SARS-CoV S glycoprotein atomic model (cyan, PDB ID: 5xlr) is fitted into each 3D density map. Outer surface: left. Central section; right. (TIF) [file ppat.1007236.s002.tif]

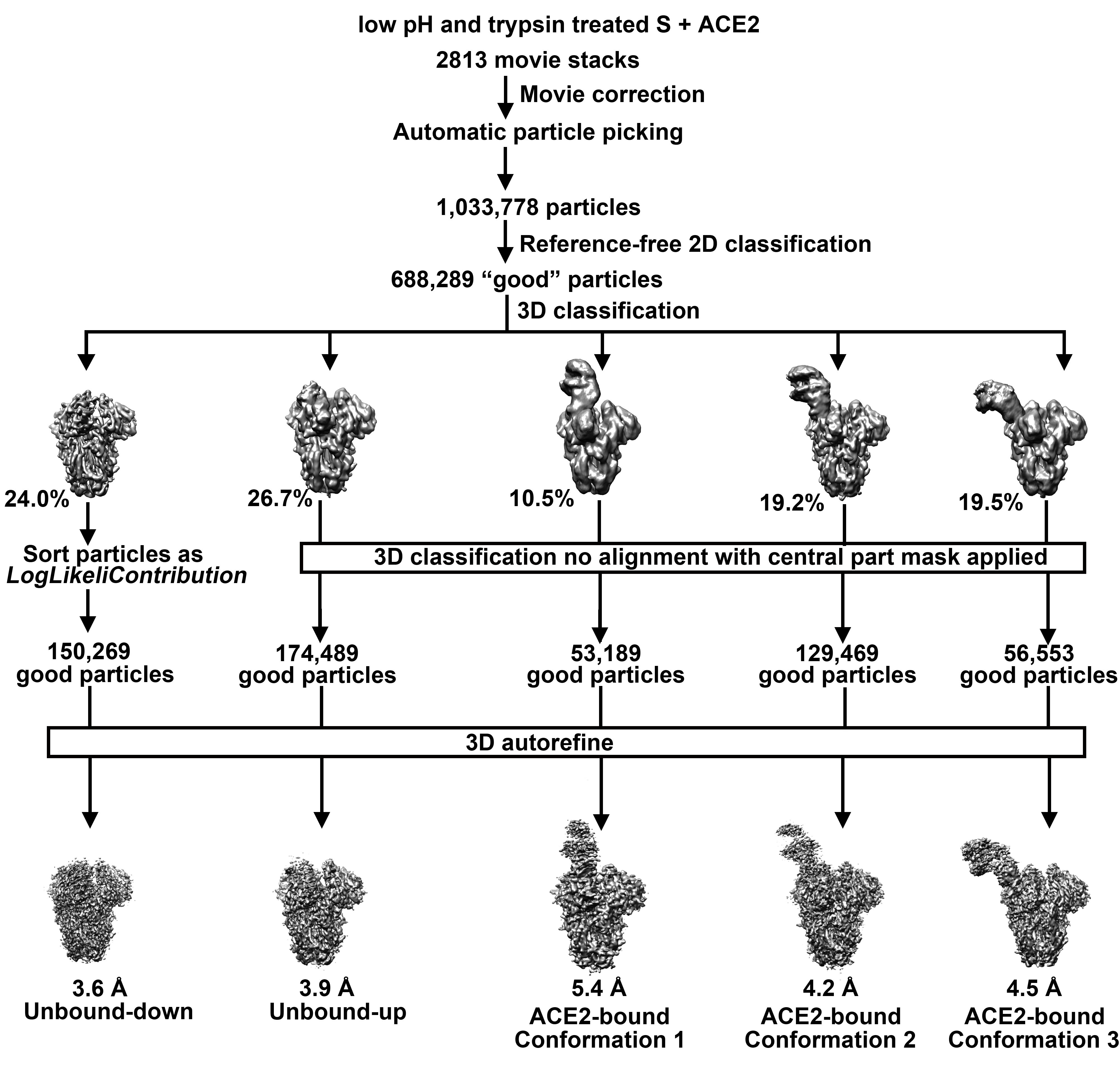

Supplement: S3 Fig — See Materials and Methods for details. (TIF) [file ppat.1007236.s003.tif]

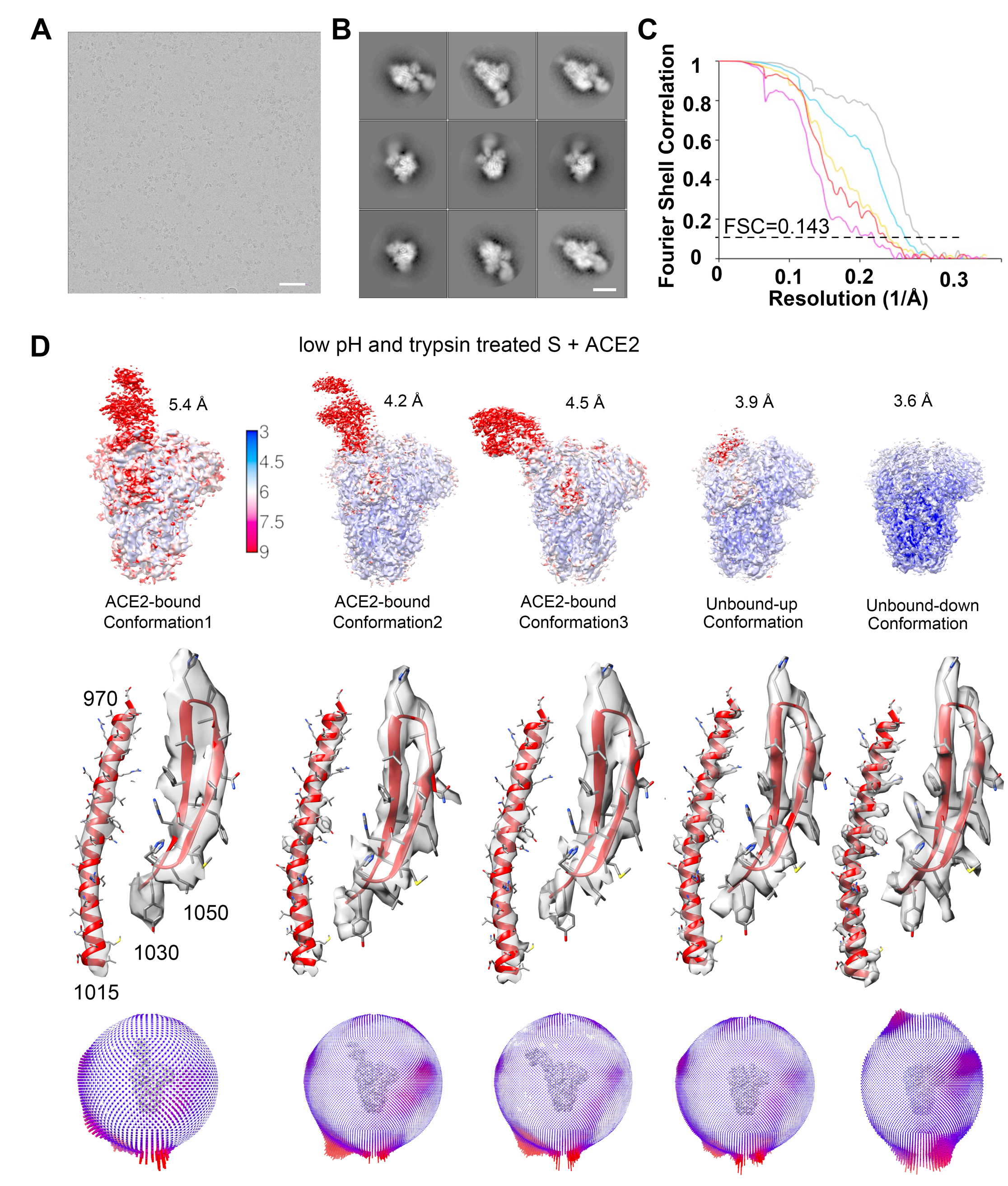

Supplement: S4 Fig — (A-B) A representative raw micrograph (A) and representative 2D class averaged images (B) of the complex. Scale bar in (A): 50 nm. Scale bar in (B): 10 nm. (C) Fourier shell correlation (FSC) curves of the 3D reconstructions. ACE2-bound conformation 1: pink, ACE2-bound conformation 2: yellow, ACE2-bound conformation 3: red, unbound-up conformation: cyan, unbound-down conformation: grey. (D) Local resolution maps, partial maps, and particle orientation distributions of the 3D reconstructions. From left to right: ACE2-bound conformation 1, ACE2-bound conformation 2, ACE2-bound conformation 3, unbound-up and unbound-down S conformation. Up: local resolution maps of the 3D reconstructions; middle: cryo-EM densities of a selected representative region; bottom: particle orientation distributions of the 3D reconstructions shown around the corresponding EM map. (TIF) [file ppat.1007236.s004.tif]

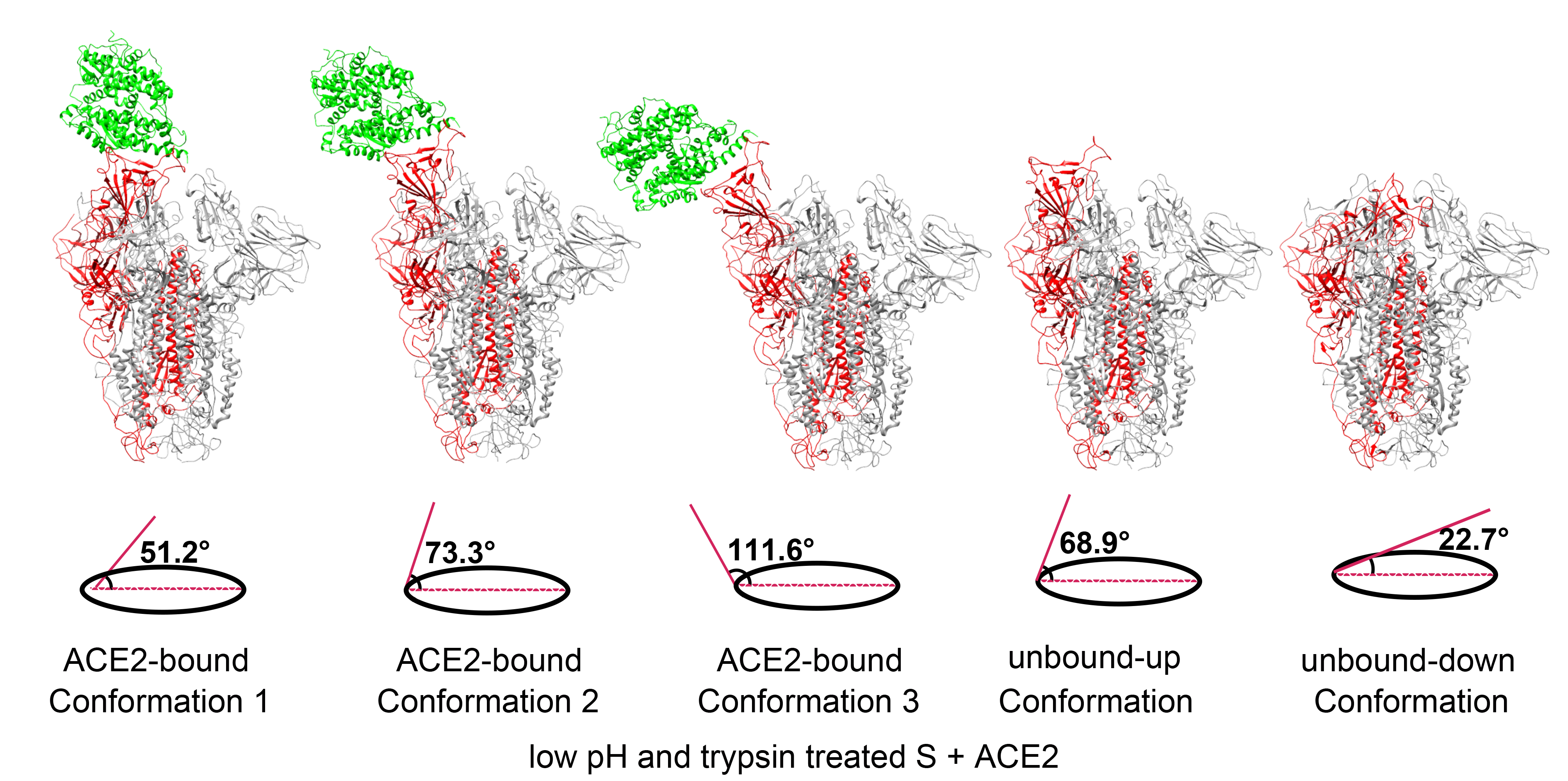

Supplement: S5 Fig — From left to right: ribbon diagrams showing the atomic models of the ACE2-bound conformation 1, the ACE2-bound conformation 2, the ACE2-bound conformation 3,unbond-up and unbound-down conformations of the SARS-CoV S glycoprotein after trypsin cleavage and low pH treatment, respectively. ACE2 binding monomer is colored red and the bound ACE2 is colored green. The angle between the long axes of the CTD1 and the horizontal plane is shown at the bottom of each structure. (TIF) [file ppat.1007236.s005.tif]

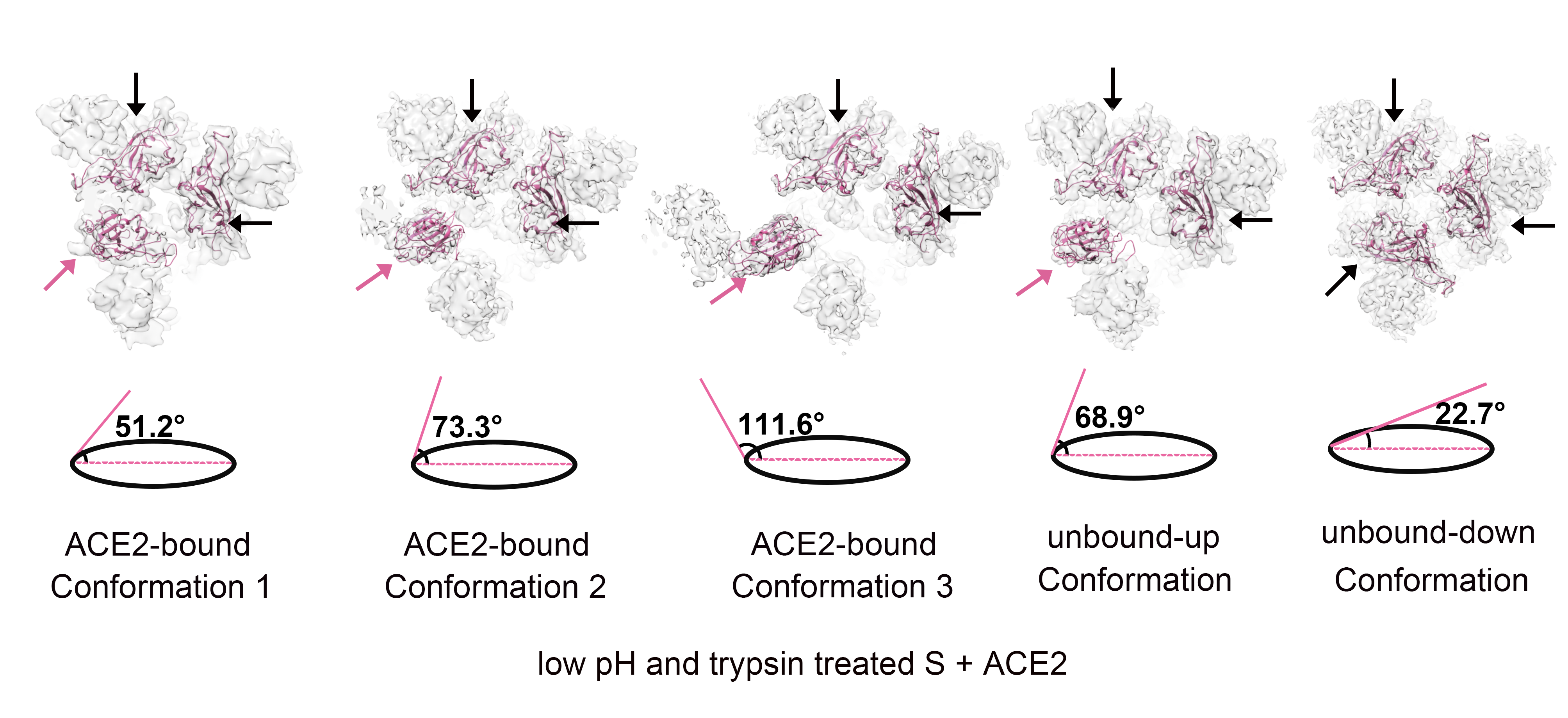

Supplement: S6 Fig — From left to right: surface shadowed diagrams showing the top views of the ACE2-bound conformation 1, the ACE2-bound conformation 2, the ACE2-bound conformation 3,unbond-up and unbound-down conformations. The CTD1s are colored pink. The black arrows indicate the “down” CTD1s, the pink arrows indicate the “up” CTD1s. The angle between the long axes of the CTD1 and the horizontal plane is shown at the bottom of each conformation. (TIF) [file ppat.1007236.s006.tif]

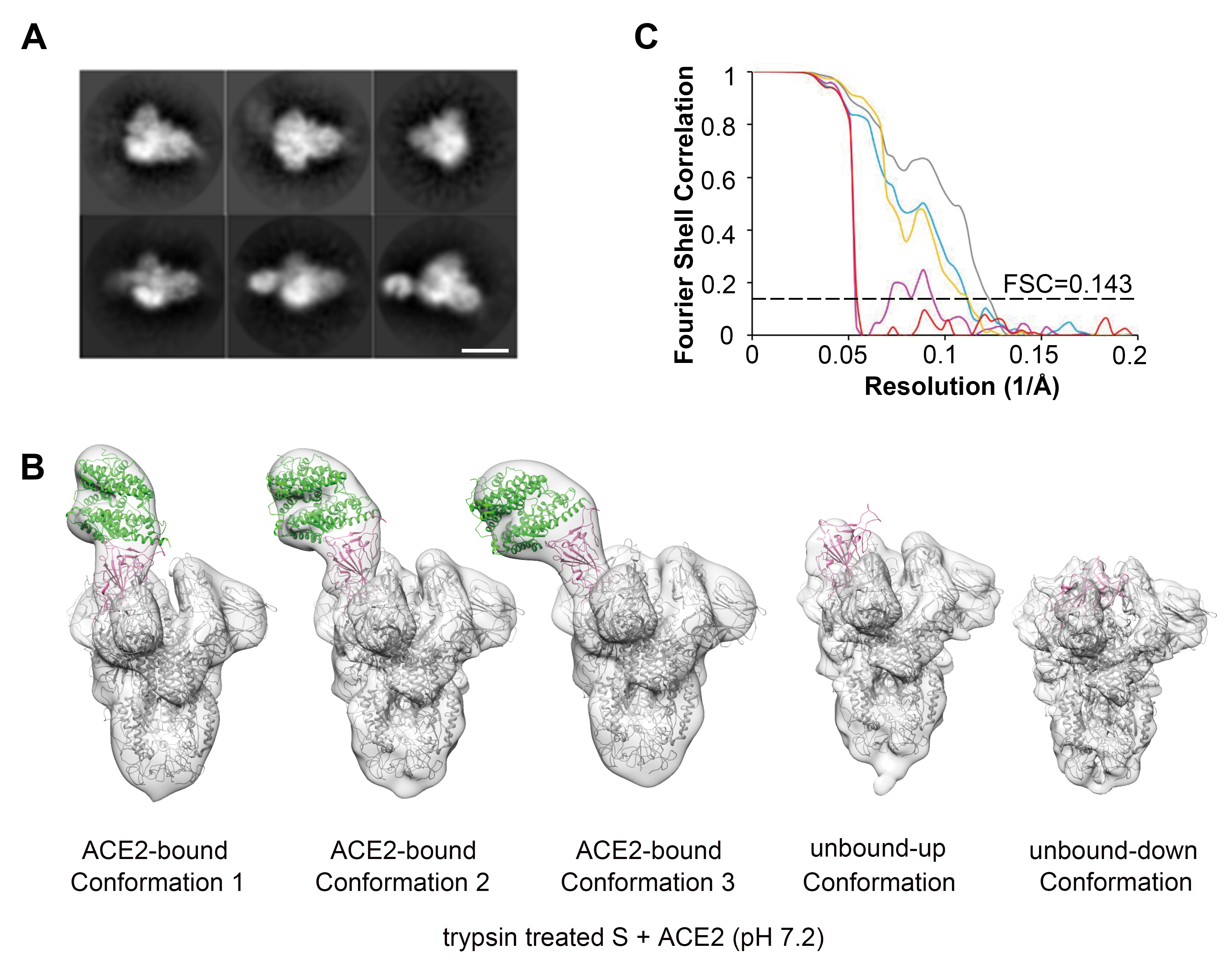

Supplement: S7 Fig — (A) 2D class averaged images of the trypsin treated S-ACE2 complex. Scale bar: 10 nm. (B) 3D density maps of the ACE2-bound conformation 1, ACE2-bound conformation 2, ACE2-bound conformation 3, unbound-up, and unbound-down conformations (from left to right). (C) Fourier shell correlation (FSC) curves of the 3D reconstructions. ACE2-bound conformation 1: pink, ACE2-bound conformation2: yellow, ACE2-bound conformation 3: red, unbound-up conformation: cyan, unbound-down conformation: grey. (TIF) [file ppat.1007236.s007.tif]

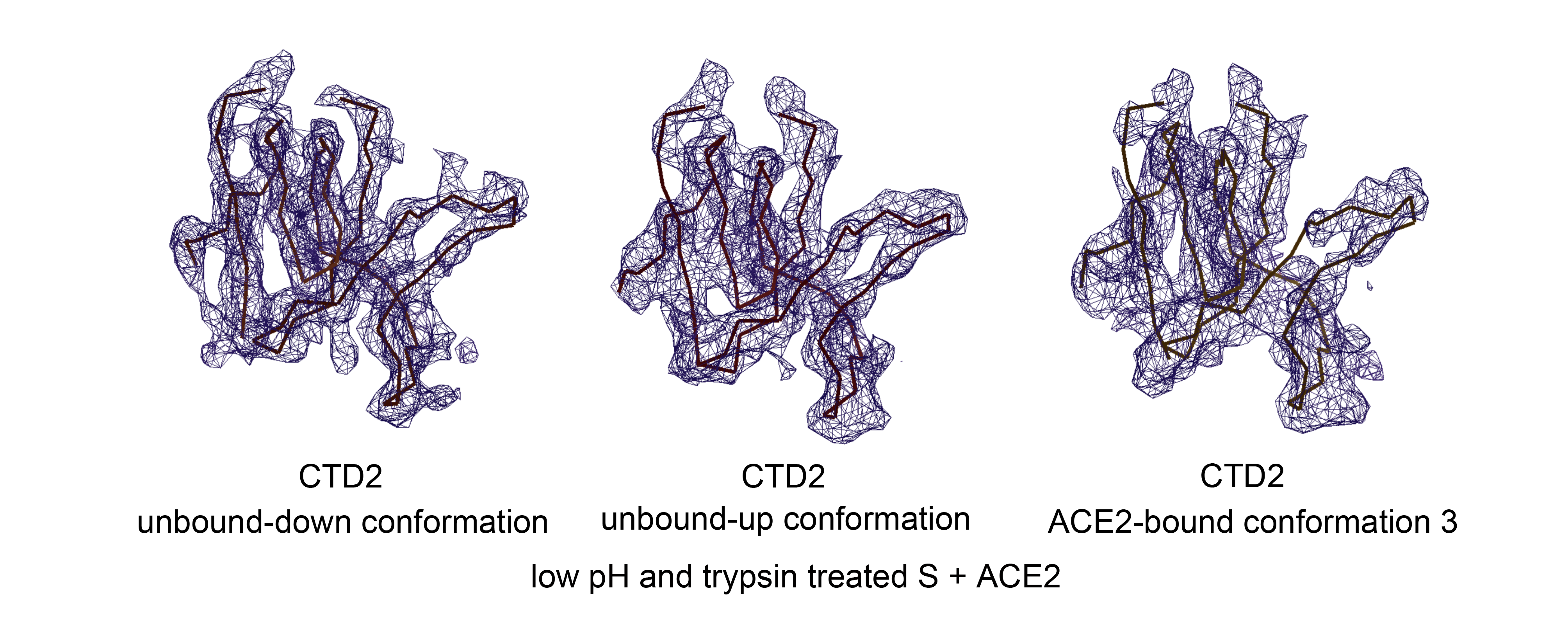

Supplement: S8 Fig — From left to right: unbound-down CTD2 (contouring level: 8 σ), unbound-up CTD2 (contouring level: 8 σ) and ACE2-bound conformation3 CTD2 (contouring level: 8 σ). The Cα backbone of the CTD2 is shown in each density map. (TIF) [file ppat.1007236.s008.tif]

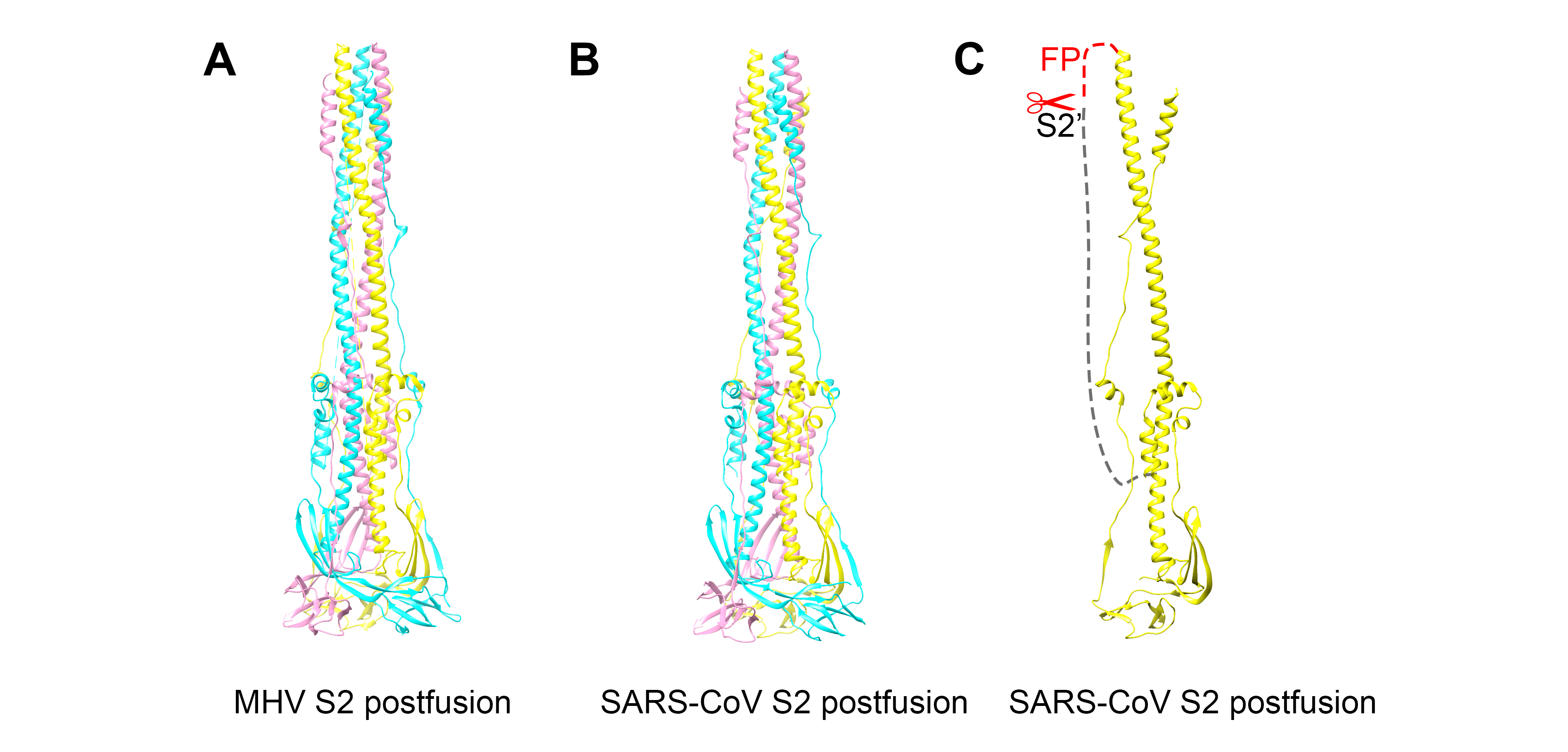

Supplement: S9 Fig — (A) Model of the post-fusion MHV S2. The three protomers are colored pink, yellow and cyan, respectively. (B) Model of the SARS-CoV post-fusion S2 obtained through homology modeling using the model in “A”. (C) Model of the SARS-CoV post-fusion S2 showing the possible locations of the S2’ cleavage site and the fusion peptide (FP, colored red). (TIF) [file ppat.1007236.s009.tif]

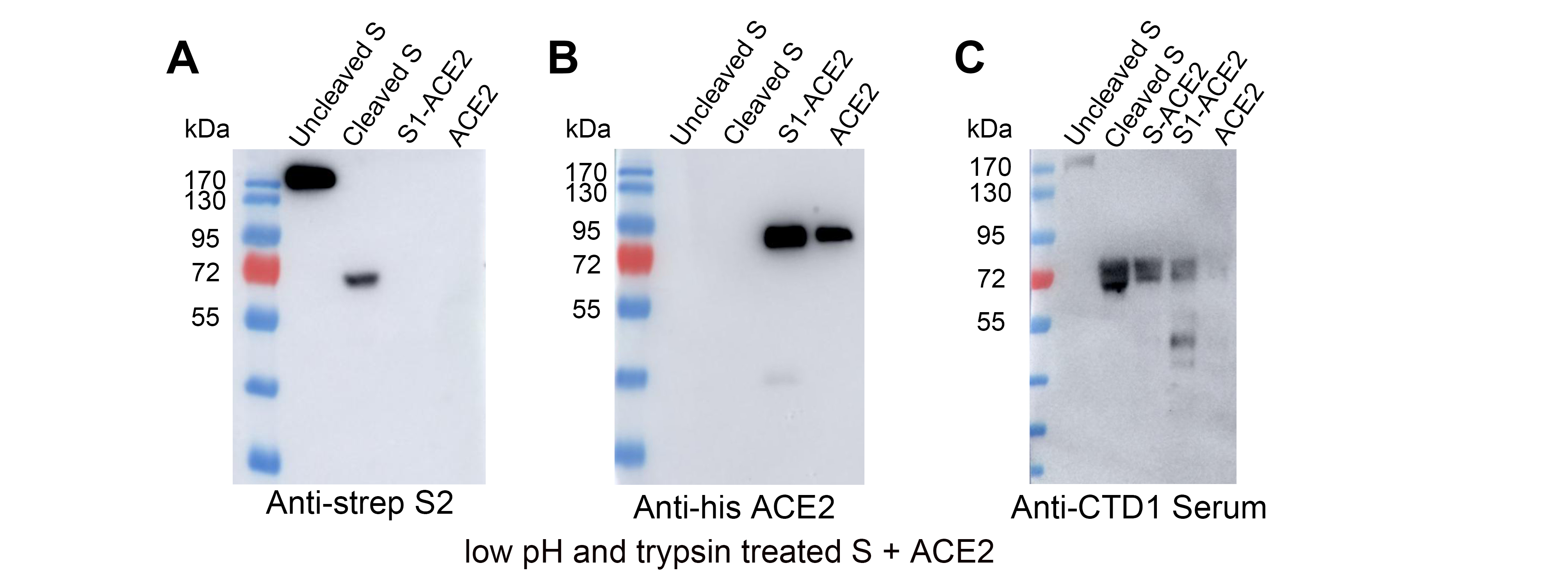

Supplement: S10 Fig — Samples are related to Fig 4B. The C-terminus of the S2 subunit contains a strep tag. ACE2 has a C-terminal his tag. Anti-CTD1 serum is generated by immunizing the mouse with the CTD1 (residue: 327–516) of the SARS-CoV S glycoprotein. The bands are detected by using anti-strep mono-antibody (A), anti-his mono-antibody (B) or anti-CTD1 serum (C). (TIF) [file ppat.1007236.s010.tif]

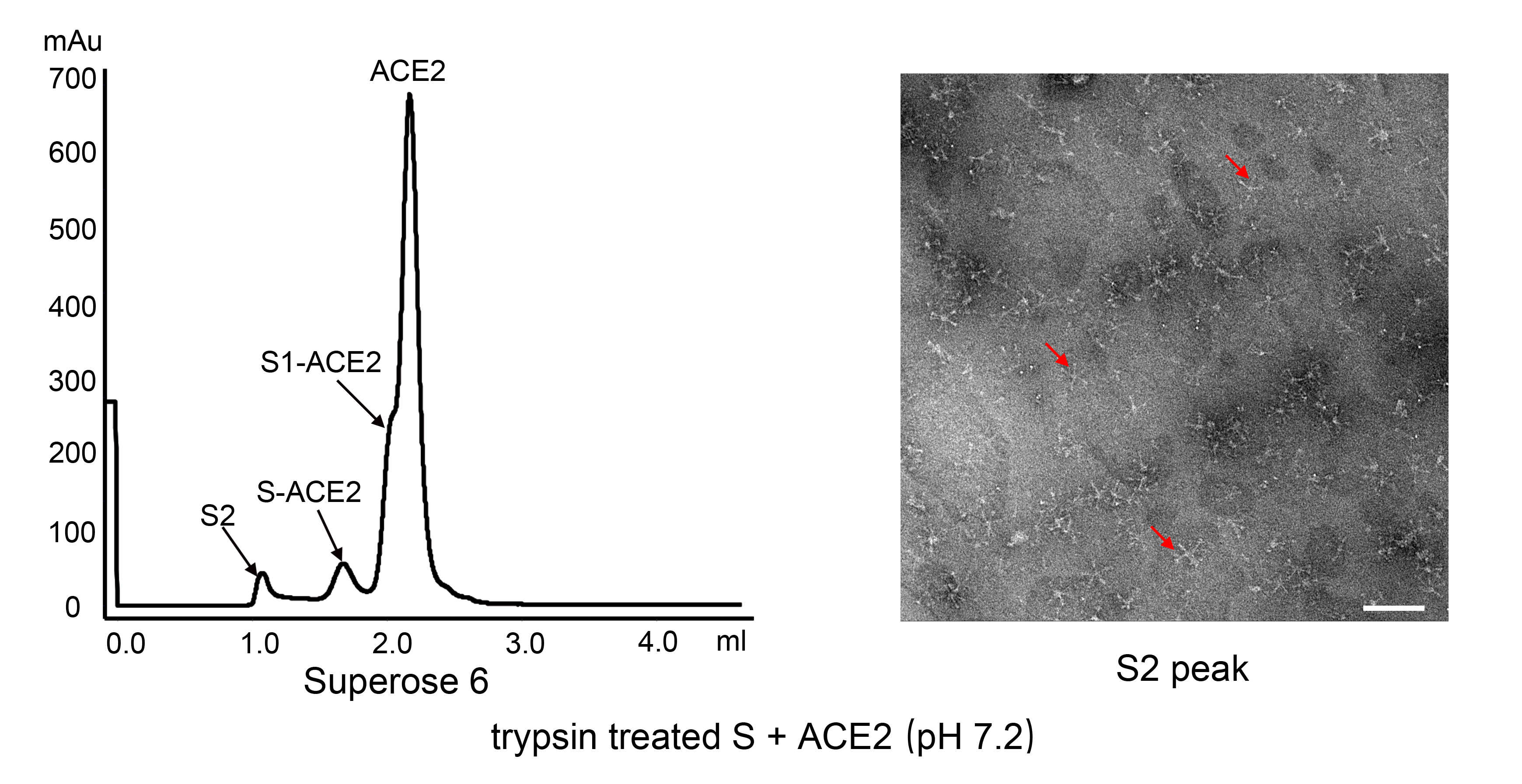

Supplement: S11 Fig — Left: size-exclusion chromatography elution profile of the trypsin treated S and ACE2 mixture at pH 7.2. Four peaks were observed, similar to that of the low pH treated sample as in Fig 3A. Right: negative staining analysis of the peak S2. Red arrows indicate the rosette-shape structures formed by the post-fusion S2 trimers. Zoom-in images are shown below the raw micrograph. Scale bar: 50 nm. (TIF) [file ppat.1007236.s011.tif]

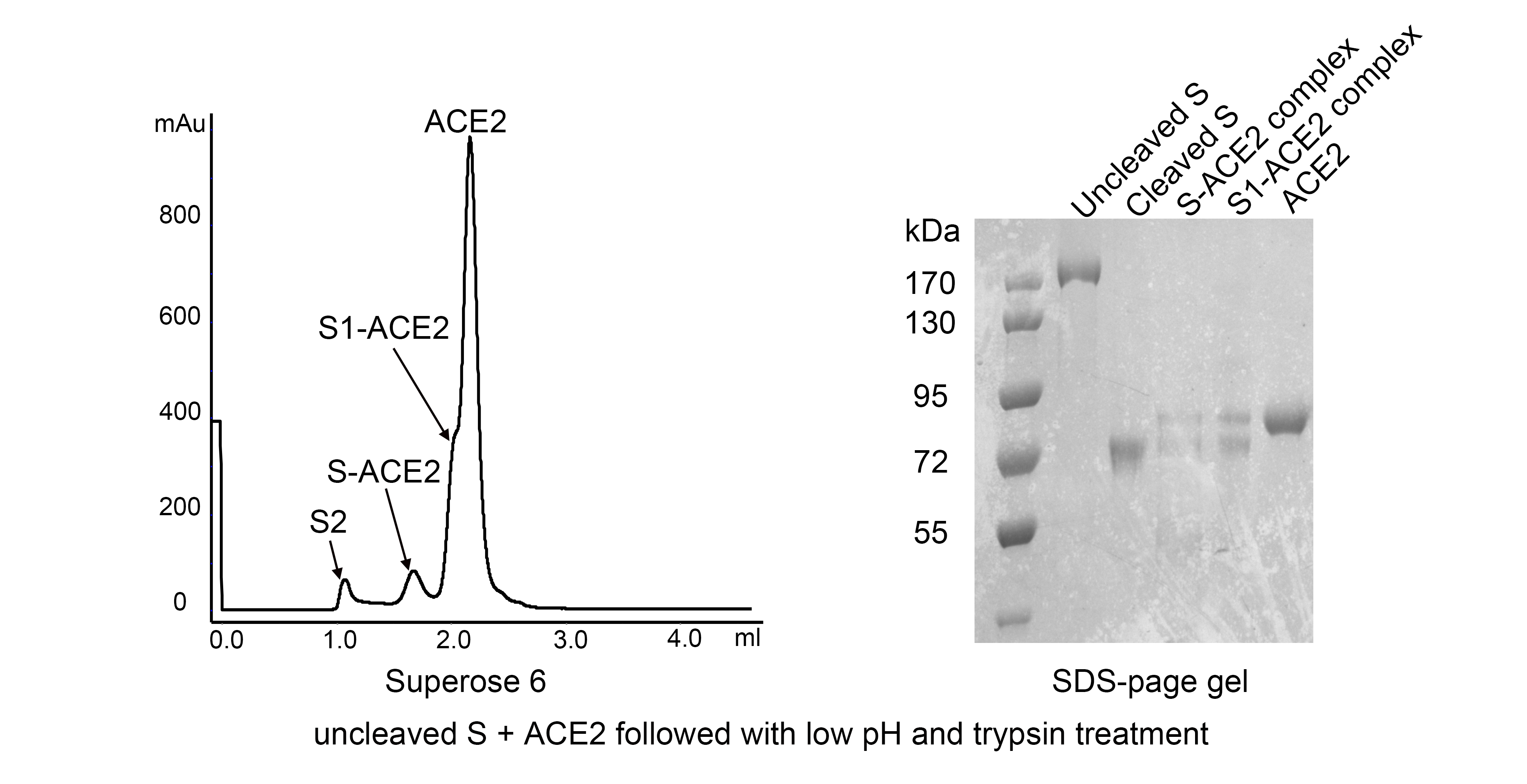

Supplement: S12 Fig — Left:Size-exclusion chromatography elution profile. Four peaks were observed, similar to the profile as in Fig 3A. Right: SDS-page analysis of the peak fractions. From left to right: marker, uncleaved S, cleaved S, S-ACE2 peak, S1-ACE2 peak and ACE2 peak. (TIF) [file ppat.1007236.s012.tif]

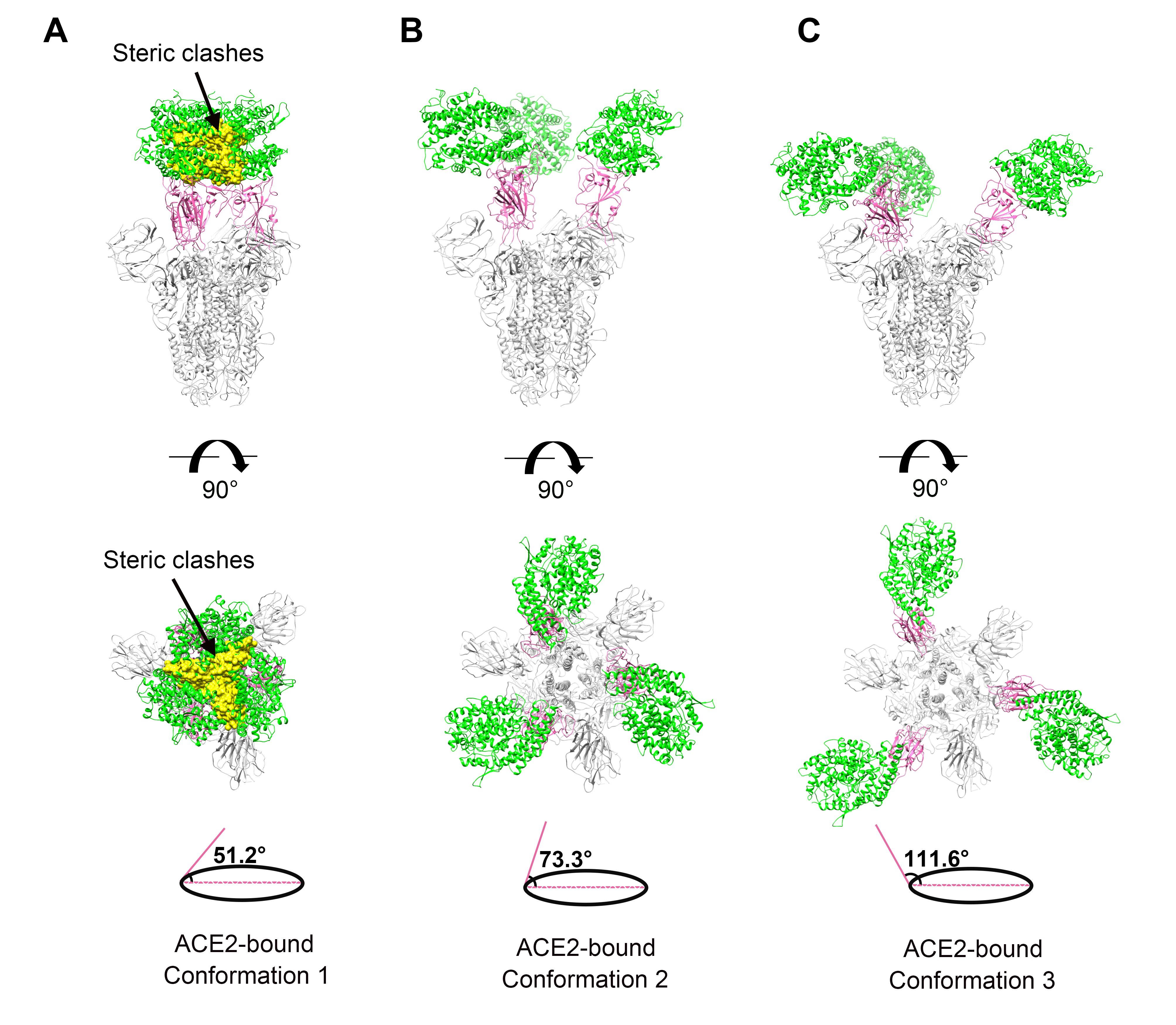

Supplement: S13 Fig — (A) Three conformation 1 “up” CTD1s binding ACE2s. The CTD1s are colored pink. Three ACE2s are colored green. The volume of the steric clashes between two neighboring monomers is 9,406 Å3 and is colored yellow. (B) Three conformation 2 “up” CTD1s binding three ACE2s. (C) Three conformation 3 “up” CTD1s binding three ACE2s. The CTD1s and ACE2 are colored the same as in “A”. No steric clash was observed for structure models in “B” and “C”. Top: side views. Middle: top views. Bottom: the angle between the long axes of the CTD1 and the horizontal plane. (TIF) [file ppat.1007236.s013.tif]
