# Supplementary material for: Cryo-EM structure of the SARS coronavirus spike glycoprotein in complex with its host cell receptor ACE2
Source: PLoS Pathog. 2018 Aug 13;14(8):e1007236. doi: 10.1371/journal.ppat.1007236 (PMC6107290; doi:10.1371/journal.ppat.1007236)
Supplement: S1 Table — (DOCX) [file ppat.1007236.s014.docx]

**S1 Table. 3D Classification statistics of different conformational states of the S-ACE2 complex**

| Sample | Cleaved and low pH treated spike + ACE2 | | | | | Cleaved spike + ACE2 | | | | |
| --- | --- | --- | --- | --- | --- | --- | --- | --- | --- | --- |
| Conformational states | ACE2-bound | | | Unbound-up | Unbound-down | ACE2-bound | | | Unbound-up | Unbound-down |
|  | Conformation  1 | Conformation  2 | Conformation  3 |  |  | Conformation  1 | Conformation  2 | Conformation  3 |  |  |
| Total particles | 688,289 | | | | | 224,052 | | | | |
| Particle number of each class | 72,265 | 132,314 | 134,192 | 184,006 | 165,512 | 26,217 | 46,321 | 23,029 | 74,553 | 53,932 |
| Particle percentage | 10.5% | 19.2% | 19.5% | 26.7% | 24.0% | 11.7% | 20.6% | 10.3% | 33.3% | 24.1% |
