# Supplementary material for: Cryo-EM structure of the SARS coronavirus spike glycoprotein in complex with its host cell receptor ACE2
Source: PLoS Pathog. 2018 Aug 13;14(8):e1007236. doi: 10.1371/journal.ppat.1007236 (PMC6107290; doi:10.1371/journal.ppat.1007236)
Supplement: S2 Table — (DOCX) [file ppat.1007236.s015.docx]

**S2 Table. Cryo-EM data collection and image processing statistics**

(A)

| Sample | Cleaved and low pH treated spike + ACE2 (S-ACE2) | | | | | S1-ACE2 | S2-rosette |
| --- | --- | --- | --- | --- | --- | --- | --- |
| Micrographs | 2813 | | | | | 450 | 524 |
| Conformational states | ACE2-bound | | | Unbound-up | Unbound-down |  | Post-fusion |
|  | Conformation 1 | Conformation 2 | Conformation 3 |  |  |  |  |
| Method | Cryo-EM | | | | | Cryo-EM | Cryo-EM |
| Acceleration voltage (keV) | 300 | | | | | 300 | 200 |
| Detector | K2 | | | | | K2 | Falcon II |
| Pixel size (Å) | 0.66 | | | | | 0.66 | 1.27 |
| Particles of final refinement | 53,189 | 129,462 | 56,553 | 174,489 | 150,269 | 41,289 | 7,636 |
| Symmetry | C1 | C1 | C1 | C1 | C3 | C1 | C3 |
| Resolution (Å) | 5.4 | 4.2 | 4.5 | 3.9 | 3.6 | 11.6 | 30.5 |
| B-factor (Å^2^) | -459 | -301 | -307 | -236 | -307 | -200 | -300 |
| Molprobity score | 2.43 | 2.28 | 2.37 | 2.10 | 1.94 |  |  |
| Rotamer outliers (%) | 0.82 | 0.55 | 0.79 | 0.25 | 0.40 |  |  |
| Clashscore | 16.57 | 11.74 | 15.24 | 8.02 | 6.06 |  |  |
| R.m.s. deviations |  | | | | |  |  |
| Bonds length (Å) | 0.0205 | 0.0199 | 0.0205 | 0.0072 | 0.0060 |  |  |
| Bonds angles (°) | 1.55 | 1.48 | 1.51 | 1.29 | 1.24 |  |  |
| Ramachandran plot (%) |  | | | | |  |  |
| Favored | 82.16 | 82.71 | 83.48 | 84.99 | 87.45 |  |  |
| Outliers | 0.48 | 0.42 | 0.32 | 0.57 | 0.60 |  |  |
| PDB ID | 6ACG | 6ACJ | 6ACK | 6ACD | 6ACC |  |  |
| EMDB ID | EMD-9591 | EMD-9593 | EMD-9594 | EMD-9589 | EMD-9588 | EMD-9598 | EMD-9597 |

(B)

| Sample | Cleaved spike | Cleaved and low pH treated spike | Cleaved spike + ACE2 (S-ACE2) | | | | |
| --- | --- | --- | --- | --- | --- | --- | --- |
| Micrographs | 663 | 342 | 1193 | | | | |
| Conformational states |  |  | ACE2-bound | | | Unbound-up | Unbound-down |
|  |  |  | Conformation 1 | Conformation 2 | Conformation 3 |  |  |
| Method | Cryo-EM | | | | | | |
| Acceleration voltage (keV) | 200  120  200  200  200 | | | | | | |
| Detector | Falcon II | | | | | | |
| Pixel size (Å) | 1.27 | | | | | | |
| Particles of final refinement | 29,848 | 43,918 | 26,217 | 46,321 | 23,029 | 74,553 | 53,932 |
| Symmetry | C3 | C3 | C1 | C1 | C1 | C1 | C3 |
| Resolution (Å) | 6.8 | 6.7 | 19.7 | 9.0 | 18.5 | 9.0 | 8.3 |
| B-factor (Å^2^) | -300 | -300 | -300 | -300 | -300 | -300 | -300 |
| EMDB ID | EMD-9595 | EMD-9596 | EMD-9585 | EMD-9586 | EMD-9587 | EMD-9584 | EMD-9583 |
