# Supplementary material for: Cryo-EM structure of the SARS coronavirus spike glycoprotein in complex with its host cell receptor ACE2
Source: PLoS Pathog. 2018 Aug 13;14(8):e1007236. doi: 10.1371/journal.ppat.1007236 (PMC6107290; doi:10.1371/journal.ppat.1007236)
Supplement: S3 Table — (DOCX) [file ppat.1007236.s016.docx]

**S3 Table. Structure modeling statistics of the ACE2-bound and ACE2-free SARS-CoV spikes.**

| Domains | Residue number in the construct | Model refinement restraints | Unassigned segments | | |
| --- | --- | --- | --- | --- | --- |
|  |  |  | ACE2-bound conformations | Unbound-up | Unbound-down |
| NTD | 1-318 | Refined as a rigid body  (reference PDB: 5x4s) | 1-17, 240-243 | 1-17, 240-243 | 1-17, 240-243 |
| CTD1 | 319-516 | Refined as a rigid body  (reference PDB: 2ajf) | 319-322, 513-516* | 319-322, 513-516 | / |
| CTD2 | 517-579 | secondary structure and geometry restraints | / | / | / |
| CTD3 | 580-667 | secondary structure and geometry restraints | 661-667 | 661-667 | 661-667 |
| S2 | 668-1195 | secondary structure and geometry restraints | 668-673, 812-831, 1120-1195 | 668-673, 812-831,  1120-1195 | 668-673, 812-831,  1120-1195 |
| ACE2 | 19-615 | Refined as a rigid body  (reference PDB: 2ajf) | / | N/A | N/A |

*For the models of the ACE2-bound conformations and the unbound-up conformation, the “up” CTD1 does not contain the residues of 319-322 and 513-516 while the “down” CTD1s include these segments.
